# Transient expression and purification of β-caryophyllene synthase in *Nicotiana benthamiana* to produce β-caryophyllene in vitro

**DOI:** 10.7717/peerj.8904

**Published:** 2020-04-28

**Authors:** Saraladevi Muthusamy, Ramesh R. Vetukuri, Anneli Lundgren, Suresh Ganji, Li-Hua Zhu, Peter E. Brodelius, Selvaraju Kanagarajan

**Affiliations:** 1 Department of Chemistry and Biomedical Sciences, Linnaeus University, Kalmar, Sweden; 2 Department of Plant Breeding, Swedish University of Agricultural Sciences, Alnarp, Sweden

**Keywords:** *Artemisia annua*, Sesquiterpene synthase, AaCPS1, Phylogenetics, Terpenoids, *Nicotiana benthamiana*, Transient expression, β-caryophyllene synthase

## Abstract

The sesquiterpene β-caryophyllene is an ubiquitous component in many plants that has commercially been used as an aroma in cosmetics and perfumes. Recent studies have shown its potential use as a therapeutic agent and biofuel. Currently, β-caryophyllene is isolated from large amounts of plant material. Molecular farming based on the *Nicotiana benthamiana* transient expression system may be used for a more sustainable production of β-caryophyllene. In this study, a full-length cDNA of a new duplicated β-caryophyllene synthase from *Artemisia annua* (*AaCPS1*) was isolated and functionally characterized. In order to produce β-caryophyllene in vitro, the *AaCPS1* was cloned into a plant viral-based vector pEAQ-*HT*. Subsequently, the plasmid was transferred into the *Agrobacterium* and agroinfiltrated into *N. benthamiana* leaves. The *AaCPS1* expression was analyzed by quantitative PCR at different time points after agroinfiltration. The highest level of transcripts was observed at 9 days post infiltration (dpi). The AaCPS1 protein was extracted from the leaves at 9 dpi and purified by cobalt–nitrilotriacetate (Co-NTA) affinity chromatography using histidine tag with a yield of 89 mg kg^−1^ fresh weight of leaves. The protein expression of AaCPS1 was also confirmed by sodium dodecyl sulfate-polyacrylamide gel electrophoresis (SDS-PAGE) and western blot analyses. AaCPS1 protein uses farnesyl diphosphate (FPP) as a substrate to produce β-caryophyllene. Product identification and determination of the activity of purified AaCPS1 were done by gas chromatography–mass spectrometry (GC–MS). GC–MS results revealed that the AaCPS1 produced maximum 26.5 ± 1 mg of β-caryophyllene per kilogram fresh weight of leaves after assaying with FPP for 6 h. Using AaCPS1 as a proof of concept, we demonstrate that *N. benthamiana* can be considered as an expression system for production of plant proteins that catalyze the formation of valuable chemicals for industrial applications.

## Introduction

Terpenoids represent one of the largest and most diverse class of secondary metabolites produced in plants (Dudareva et al., 2006). Recently, terpenoids have been attracting considerable attention due to their use as solvents and diluting agents for dyes and varnishes, ingredients in fragrances, flavors in foods and as pharmaceutical compounds (Wagner & Elmadfa, 2003). All terpenoids are formed from the same basic five-carbon unit, that is, the isoprene unit and are classified by the number of isoprene units they contain (Newman & Chappell, 1999). The terpenoids that are derived from three isoprene units are known as sesquiterpenes, which is the largest group of terpenoids in plants (Nagegowda, 2010). β-Caryophyllene is a major volatile organic bicyclic sesquiterpene that has traditionally been used to provide a woody, spicy aroma and fragrance to cosmetics and perfumes. Studies have suggested that β-caryophyllene also possesses anti-inflammatory (Andrade-Silva et al., 2016), antioxidant (Basha & Sankaranarayanan, 2016), anticonvulsant (Oliveira et al., 2016), anti-alcoholism (Bahi et al., 2014), antinociceptive (Katsuyama et al., 2013), anxiolytic, antidepressant (Bahi et al., 2014), antiallodynic (Segat et al., 2017), cardioprotective, hepatoprotective, gastroprotective, nephroprotective (Machado et al., 2018) and anticarcinogenic (Dahham et al., 2015) activities.

β-Caryophyllene is one of the most widely distributed sesquiterpene in the plant kingdom. Plants emit β-caryophyllene as a plant defense substance against herbivores. β-Caryophyllene can directly and/or indirectly repel herbivores to protect the plant by attracting natural enemies of herbivores (Köllner et al., 2008; Wang et al., 2015). Plants release β-caryophyllene that may mediate antagonistic interactions with bacterial pathogens (Huang et al., 2012). In *Zingiber nimmonii*, caryophyllene-rich rhizome oil showed significant inhibitory activity against bacteria and fungi (Sabulal et al., 2006).

Although β-caryophyllene has diverse roles in different industrial applications and exhibits antibacterial and antifungal activities, it cannot be extracted from natural sources in a sustainable way, either due to its low concentrations in the raw material or due to poor recovery yields (Quispe-Condori et al., 2008). Also, the complexity of the chemical synthesis and high cost make the commercial production of β-caryophyllene infeasible and uneconomical (Topal et al., 2008). Therefore, interest has been toward developing alternative sustainable technologies to produce β-caryophyllene in a large scale (Yang & Nie, 2016).

During the last decades, the development of bio-based molecules to replace synthetic/chemical-based resources has been constantly increasing. Although many microorganisms can provide the substrate farnesyl diphosphate (FPP), they cannot produce β-caryophyllene due to a lack of β-caryophyllene synthase (CPS). Several microorganisms (*Escherichia coli* and *Synechocystis* sp.) have been engineered with *A. annua* CPS to produce β-caryophyllene (Cai et al., 2002; Reinsvold et al., 2011; Wu, Liu & Davis, 2018). However, formation of inclusion bodies in *E. coli* coupled with nonspecific product formation by the terpene synthases (Picaud et al., 2006; Picaud, Olsson & Brodelius, 2007), demands an alternative platform for the expression of sesquiterpene synthases, such as CPS.

Production of β-caryophyllene using an enzyme produced in plants offers great potential in addressing such issues and thus has been the focus of our study. As production of sesquiterpenes by plant-derived enzymes in plant expression system has demonstrated high selectivity toward a specific product (Kanagarajan et al., 2012a), plant-based production of CPS is expected to be an attractive alternative to traditional methods. With the advances in plant biotechnology made in the past decades, plants have been used as an alternative for microbial production systems due to ingenuous and its unlimited scalability. Also, plants have a simple and inexpensive growth requirement, are free from human pathogens and offers minimal/easy handling processes of protein production.

So far, different *Nicotiana* species have been used as major host systems for production of recombinant proteins through transient gene expression due to their robust growth rate, cost effective production and simplicity for genetic engineering, high soluble proteins compared to other model crops. *Nicotiana* species have been functioning as efficient host systems for production of industrially valuable proteins (Tremblay et al., 2010). In *N. benthamiana*, FPP synthase (FDS) and 3-hydroxy-3-methylglutaryl CoA reductase (HMGR) were transiently expressed to improve the availability of the substrate of sesquiterpene synthases (Van Herpen et al., 2010). Also, we have expressed two sesquiterpene synthases, amorpha-4,11-diene synthase (ADS) and *epi*-cedrol synthase (ECS) by transient expression using agroinfiltration into *N. benthamiana* (Kanagarajan et al., 2012a).

In the present study, we have overexpressed β-caryophyllene synthase (*AaCPS1*) gene, an isoform of *CPS* from *A. annua* in *N. benthamiana* by an *Agrobacterium*-mediated transient expression approach to produce β-caryophyllene in vitro.

## Materials and Methods

### Plant material

Seeds of *Artemisia annua* L. cv Anamed were obtained from Anamed, Germany (http://www.anamed.net). After sowing, three weeks old seedlings of *A. annua* and *N. benthamiana* were transplanted into individual pots and maintained at 23 ± 1 °C with a 16 h light/8 h dark photoperiod in a greenhouse.

### Bacterial strains

*Escherichia coli* (Novablue, Novagen) was used for cloning and *Agrobacterium tumefaciens* strain, LBA4404 was used for plant infiltration. The bacteria were grown in Luria–Bertani media with appropriate antibiotics at 36 ± 1 °C and 25 ± 1 °C, respectively.

### Gene isolation and phylogenetic analysis

Leaves of *A. annua* plants were wounded at vegetative stage by cutting (3–5 mm) along the midrib with a razor blade. After 12 h the wounded leaves were collected and RNA extraction was performed using Purelink^™^ Plant RNA Reagent kit (Invitrogen, Carlsbad, CA, USA) according to the manufacturer’s instructions. The RNA was treated using DNase I (Fermentas, St. Leo-Roth, Germany) and was reverse transcribed using RevertAid™H Minus-MuLV reverse transcriptase (Fermentas, St. Leo-Roth, Germany). The RNA was removed from the first strand cDNA by RNase treatment using RNase H (Fermentas, St. Leo-Roth, Germany) according to the manufacturer’s instructions.

To isolate the full length ORF of the *AaCPS1* gene from cDNA from *A. annua* leaves, PCR was performed with a gene-specific forward primer (5′-ATGGACATGCCTGCAAAAG-3′) that was designed based on sequence information obtained from our previous study (Wang et al., 2013) and nucleotide sequences of conserved region of previously reported *CPS* reverse primer (5′-TTATATAGGTATAGGATGAAC-3′) of *A. annua* (Cai et al., 2002). The PCR product was separated on 1.2% (w/v) agarose gels, cloned into pJET vector and then sequenced. Verification of the cDNA sequence and protein sequence, theoretical isoelectric point (pI), and predicted molecular weight (MW) analyses of the *AaCPS1*, were performed using ExPASy Proteomic tools (https://www.expasy.org/tools/). The stability of *AaCPS1* protein was analyzed by the ProtParam tool (http://web.expasy.org/program/). DNA and protein sequence alignments were performed using the CLC Main Workbench 5.7.1 (CLC Bio, Århus, Denmark). The nucleotide sequence obtained for *AaCPS1* was deposited in the GenBank database and the accession number is KC118534.1. The protein sequences were also searched for secretion signal peptides using SignalP (http://www.cbs.dtu.dk/services/SignalP/).

To investigate the evolutionary relationship of the *CPS* genes in *A. annua* and CPSs from a few plant species of the Asteraceae family, a phylogenetic analysis was carried out. We have used the full-length protein sequences of a total of 13 CPSs and six other sesquiterpene synthases across the Asteraceae family. To perform phylogeny reconstruction, we performed multiple protein sequence alignments of the 19 sesquiterpene synthases using ClustalW. A phylogenetic tree was constructed by the neighbor-joining algorithm using MEGA7 by using the *p*-distance substitution model (Kumar, Stecher & Tamura, 2016). The bootstrap statistical analysis was performed using 1,000 replicates to evaluate the statistical significance of each node.

### Plant expression vector construction

**Figure 1:**
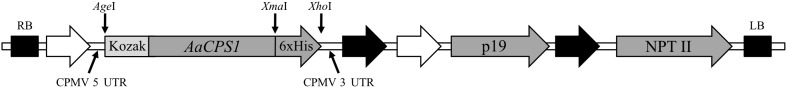
Schematic representation of the pEAQ-*HT* vector containing β-caryophyllene synthase of *Artemisia annua* (*AaCPS1*) gene cassette, used for transient expression in *N. benthamiana*. The T-DNA region of the illustrated vector features include: RB, T-DNA right border; LB, T-DNA left border; White arrows, CaMV duplicated 35S promoter. Black arrows, CaMV poly A signal sequence/terminator.

A full-length *AaCPS1* gene was amplified from pJET vector by PCR using gene specific primers; forward primer: 5′-AACCGGTGCCACCATGGACATGCCTGCAAAAG-3′ with an *Age*I restriction site and Kozak consensus sequence (GCCACC) (Kozak, 1989) at the 5′ end; reverse primer: 5′-AGCGCCCGGGTATAGGTATAGGATGAACGAGC-3′ with a *Xma*I restriction site 3′ end. The PCR amplified product was purified by the PCR purification kit (Fermentas, Waltham, MA, USA), ligated into pJET vector and transformed into *E. coli* competent cells by the heat shock method according to the manufacturer’s instructions. The construct was verified by DNA sequencing. Subsequently, the construct was digested with *Age*I and *Xma*I restriction enzymes and then subcloned into a cowpea mosaic virus-based plant expression vector, pEAQ-*HT* (Sainsbury, Thuenemann & Lomonossoff, 2009), carrying a sequence encoding a 6× His-tag at the 3′-end, resulting in the construct of pEAQ-*HT*-AaCPS1 (Fig. 1).

### Transient expression *in* *N. benthamiana* leaves

The pEAQ-*HT-*AaCPS1 vector along with the pJL3:P19 vector, harboring posttranscriptional gene silencing gene suppressor protein, P19 from tomato bushy stunt virus (TBSV) was separately transformed into *A. tumefaciens* strain, LBA4404 by the freeze thaw method and confirmed by PCR. The overnight grown *Agrobacterium* culture with OD_600_ of about 1.2–1.4 was harvested by centrifugation for 20 min at 2,000*g*. Cell pellet was suspended in 10 mM MES buffer, pH 5.5, containing 10 mM 2-*N*-morpholino-ethanesulfonic acid, 10 mM MgCl_2_, and 100 µM acetosyringone to a final OD_600_ of 0.5. Cells were incubated at room temperature for 2–4 h. For co-infiltration of pEAQ-*HT-*AaCPS1 and pJL3:P19, equal volumes of the *Agrobacterium* cultures were mixed and infiltrated into leaves of *N. benthamiana* as described previously (Kanagarajan et al., 2012b). Plant leaves were harvested at six different time points (0, 3, 6, 9, 12 and 15 days) after infiltration and samples were processed immediately or frozen in liquid nitrogen and stored at −80 °C until further use.

### Relative gene expression using real-time quantitative reverse transcriptase-polymerase chain reaction (qPCR)

RNA isolation from leaf samples at 0, 3, 6, 9, 12- and 15-days post infiltration (dpi), first strand cDNA synthesis and qPCR were performed as previously described (Olofsson et al., 2011). Amplification efficiency of primers was determined by measuring serial dilutions of 1 μg of cDNA from control and *Agrobacterium* infiltrated leaf samples in triplicates. Then, qPCR was performed using specific primers (Cai et al., 2002) on an ABI Prism^®^ 7500 Sequence Detection System (Applied Biosystems, Foster City, CA, USA). Each reaction was performed in a total volume of 20 μl consisting of one μl of first strand cDNA as template, 2 pmol each of forward (AaCPS1 RT FOR 5′-GGGAAAAGAGGGAAAAGCACATC-3′) and reverse (AaCPS1 RT REV 5′-GCTGCTTACAAACGCAACTGAC-3′) primers with 10 μl Power SYBR^®^ Green PCR master mix (Applied Biosystems, Foster City, CA, USA) using the program: 50 °C for 2 min, 95 °C for 10 min followed by 40 cycles of 95 °C for 15 s, 60 °C for 1 min and dissociation stage at 95 °C for 15 s, 60 °C for 1 min, 95 °C for 15 s. The melting curve analysis was carried out to determine the specificity of primer pair in the reaction. To compare transcripts at different time points, all qPCR reactions were performed with equal quantity of total RNA. All qPCRs reactions were performed in three technical replicates for each of three biological replicates along with negative controls (in which reaction mixture did not contain template DNA). The *ACTIN* (forward primer 5′-GGTCGTGACCTCACTGATAGTTTG-3′ and reverse primer 5′-GCTGTGGTAGTGGATGAGTAACC-3′) and *elongation factor 1* (*EF1*) (forward primer 5′-GATTGGTGGTATTGGAACTGTC-3′ and reverse primer 5′-AGCTTCGTGGTGCATCTC-3′) genes were used as reference genes for normalization of gene expression. The efficiency of qPCR was determined with the slope of a linear regression model (Pfaffl, 2001) and the Cycle threshold (Ct) values were used for further analysis. Relative gene expression levels were calculated using the REST 2009 software version. 2.0.13 (Pfaffl, Horgan & Dempfle, 2002). Relative expression was determined by comparing the Ct value of transcripts of different times (3, 6, 9, 12 and 15 dpi) with the sample having lowest gene expression.

### Protein extraction and quantification

*Agrobacterium* infiltrated *N. benthamiana* leaves were grounded with liquid nitrogen. Then, total soluble protein (TSP) from grounded leaves was extracted using 1.5 v/w of extraction buffer (20 mM sodium phosphate buffer, pH 7.2, containing 0.5 M NaCl, 5 mM dithiothreitol (DTT), 5% polyvinylpolypyrrolidone and 0.1% protease inhibitor (P9599; Sigma, Kawasaki, Kanagawa, Japan). Protein quantification was done by BioRad protein assay (Bradford, 1976) using Bovine Serum Albumin (BSA) as a standard in spectrophotometer (ND-1000; Nanodrop^®^, Wilimgton, DE, USA). Enzyme expression efficiency was determined by sodium dodecyl sulfate-polyacrylamide gel electrophoresis (SDS-PAGE) and western blot analysis using proteins extracted at different time points.

### Protein purification

Recombinant AaCPS1 protein with carboxy (C)-terminal His-tag was purified from TSP by immobilized metal ion affinity chromatography (IMAC). TSP (100–150 mL) was passed through a five mL HiTrap Chelating HP column (GE Healthcare, Chicago, IA, USA) charged with Co^2+^. Then, the column was washed with binding buffer (20 mM sodium phosphate, pH 7.4, 0.5 M NaCl, 30 mM imidazole) to remove weakly bound proteins. AaCPS1 was eluted with elution buffer (20 mM sodium phosphate, pH 7.4, 0.5 M NaCl, 500 mM imidazole). Detection of protein in eluted fractions was done by measuring absorbance at 280 nm. Fractions having the highest concentrations of recombinant protein were pooled and desalted with PD-10 column (GE Healthcare, Chicago, IA, USA) equilibrated with assay buffer containing 20 mM Tris–HCl, pH 7.0, 20 mM MgCl_2_, 10% (v/v) glycerol and 2 mM DTT. Subsequently, purified protein in desalted fractions was quantified using the Bradford assay, and protein purity was estimated with SDS–PAGE. The purified protein was stored at −20 °C.

### SDS–PAGE and western blotting

SDS–PAGE electrophoresis was performed with TSPs (7 µg) or purified protein (0.5 µg) using NuPAGE™ 1X MES SDS Running Buffer (Invitrogen, Carlsbad, CA, USA), and NuPAGE™ 4–12% Bis/Tris gels (Invitrogen, Carlsbad, CA, USA) according to the manufacturer’s instructions using a Novex™ X-Cell II™ mini horizontal gel electrophoresis unit. The gels were stained with PageBlue™ Protein staining solution (Fermentas, St. Leo-Roth, Germany) following the manufacturer’s basic protocol or used for western blot. Proteins on the gel were electroblotted into 0.45 µm polyvinylidene fluoride (PVDF) membranes using a Novex™ X-Cell II™ Blot module following the manufacturer’s instruction. PageRuler Prestained Protein Ladder (Fermentas, St. Leo-Roth, Germany) was included in SDS–PAGE and western blots as molecular weight markers to determine the protein size. Immunoblot detection was performed using Anti-His (C-term)-HRP Antibody (Invitrogen, Carlsbad, CA, USA) according to the manufacturer’s protocol. Bound antibodies were detected by using the Amersham ECL Western Blotting Detection Reagent (GE Healthcare, Chicago, IA, USA) in accordance with the manufacturer´s procedures. Quantification of the AaCPS1 in TSPs and purification efficiency of the AaCPS1 were calculated by densitometric analysis, using ImageJ software version 1.52k (Schneider, Rasband & Eliceiri, 2012).

### Product identification and quantification

TSP from empty vector (pEAQ-*HT* and pJL3:p19) infiltrated leaves (10 µg), TSP from *N. benthamiana* leaves (10 µg), purified protein from empty vector (pEAQ-*HT* and pJL3:p19) infiltrated *N. benthamiana* leaves and purified enzyme (10 µg) were assayed in 20 mM Tris–HCl, pH 7.0, 20 mM MgCl_2_, 10% (v/v) glycerol and 2 mM DTT and 20 µM FPP in a total volume of 500 µL, overlaid with 50 µL of dodecane. The samples were incubated for 4 h at 30 °C in a water bath and one µL of sample from the dodecane phase was analyzed by gas chromatography–mass spectrometry (GC–MS) for identifying and quantifying β-caryophyllene. Samples were analyzed by capillary GC on an Agilent 6890 series gas chromatograph equipped with a HP-5MS 5% phenyl methyl silohexane capillary column (0.2 mm film thickness, 0.25 mm i.d., 30 m length; Agilent Technologies, Santa Clara, CA, USA) and an Agilent 5973 Network Mass Selective Detector. The GC injection port was operated with a carrier gas of helium at a constant flow of 0.7 mL min^−1^ with one µL splitless injection mode. The GC was performed with an injector temperature of 40 °C and the oven temperature was programed from 50 °C for 5 min, 50–200 °C at a rate of 2 °C min^−1^ and finally increased to 300 °C with 25 °C min^−1^ for 5 min. Mass spectra were measured with the mass range of m/z 34–400, at an electron voltage of 70 eV, and interface temperature of 250 °C. For all runs, a solvent delay of 4 min was allowed prior to acquisition of MS data. The β-caryophyllene and α-humulene peaks were identified by comparison of the GC–MS profile with authentic standards of β-caryophyllene (W225207; Sigma Aldrich, St. Louis, MO, USA) and α-humulene (53675; Sigma Aldrich, St. Louis, MO, USA). The β-caryophyllene peak was quantified by integration of peak areas using Enhanced Chemstation version E.02.00.493 (Agilent Technologies, Santa Clara, CA, USA). A standard curve of β-caryophyllene (5–100 ng µL^−1^) was used for the quantification of β-caryophyllene in the samples collected at different time points. Three replicates were used to quantify the absolute concentration of β-caryophyllene. Data analyses were performed using Microsoft Excel™.

## Results

### Isolation of *AaCPS1* gene and phylogenetic analysis

We successfully isolated the *AaCPS1* gene, an isoform of *CPS* from *A. annua*. The *AaCPS1* nucleotide sequence obtained from this study contained an ORF of 1,653 bp. Nucleotide sequence alignment of the *AaCPS1* gene with a previously reported and characterized *CPS* (*QHS1*) of *A. annua* (GenBank accession number, AF472361.1) indicated 98% identity (Fig. S1). The *AaCPS1* gene identified in this study, was used as a BLAST query to identify putative *CPSs* in *A. annua* using NCBI. This search rendered three putative *CPSs* of *A. annua*. The *AaCPS1* gene showed higher than 99% nucleotide sequence identity with one of the *CPS* sequences (*CPS*, GenBank accession number, PKPP01015792.1) identified in the recent genome and transcriptomic sequencing report (Shen et al., 2018), indicating that the *AaCPS1* gene is an isoform of this *CPS* gene (Fig. S2). These two genes were designated as *AaCPS1*. Moreover, through nucleotide sequence alignment of *AaCPS1* with other CPSs, *AaCPS1* gene shared 98% nucleotide sequence identity with *CPS* (GenBank accession number, PKPP01013206.1) and 91% nucleotide sequence identity with *CPS* (GenBank accession number, PKPP01009489.1) and named as *AaCPS2* and *AaCPS3*, respectively (Figs. S3 and S4).

The deduced AaCPS1 protein encoded by the coding region is 550 amino acid long. The predicted molecular mass and the calculated isoelectric point of AaCPS1 protein are 63.99 kDa and 5.65, respectively. The deduced amino acid sequence of AaCPS1 (PWA38292.1) showed no signal peptide (Fig. S5). The presence of the conserved domains in the deduced protein sequences of AaCPS1 was consistent with and similar to that of the other terpene synthase features (Figs. S5–S7). The C-terminal Mg^2+^-binding domain (residue 303–307) contained the highly conserved aspartate-rich motif (DDxxD). In addition to this motif, the conserved arginine-rich RxR motif, which was located at 35 amino acids upstream of the first DDxxD motif, was identified. The conserved motifs presented as RDR, DDTYD and NSE/DTE in all members of CPSs except AaCPS3. Multiple sequence alignment of the protein sequences of AaCPS1 with other *Artemisia* CPSs showed very high sequence similarity (87–99%) (Fig. S6).

**Figure 2:**
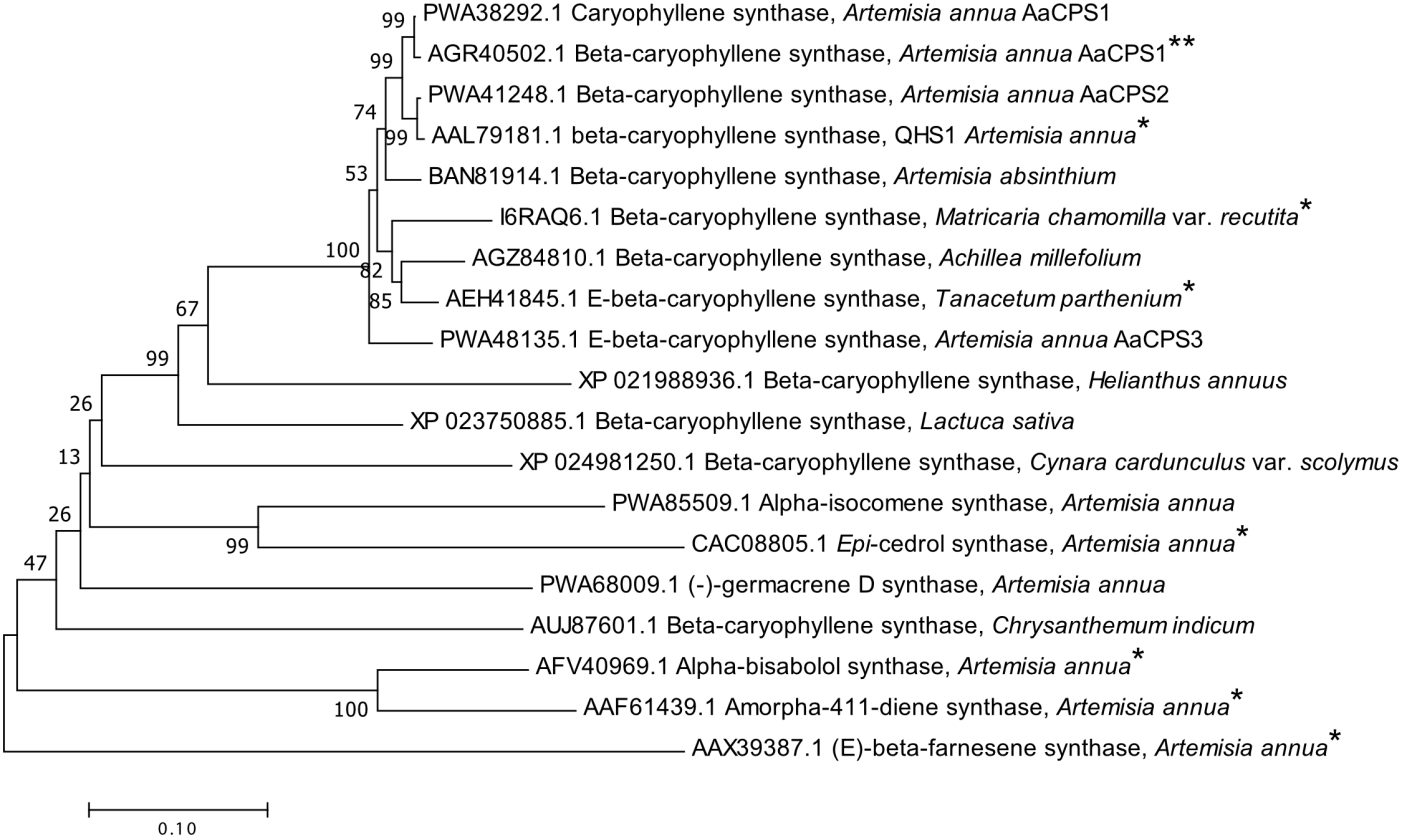
Phylogenetic analysis of β-caryophyllene synthase of *Artemisia annua* (*AaCPS1*) together with other β-caryophyllene synthases (*CPS*s) and few sesquiterpene synthases. The phylogenetic tree for *CPS*s and sesquiterpene synthases from *Artemisia* genus and different plant species belonging to the Asteraceae family were constructed using multiple alignments of deduced amino acid sequences performed using ClustalW. An unrooted neighbor‐joining (NJ) tree was generated using MEGA7. The database accession numbers are indicated before plant names. Bootstrap analysis was performed with 1,000 replicates to obtain a support value for each branch. Scale bar represents 0.05 amino acid substitutions per site. Numbers given at branches are bootstrap values. *Denotes functionally characterized proteins, **denotes protein characterized in the current study.

To elucidate the evolutionary relationship, a phylogenetic tree was constructed based on protein sequence alignment of the CPSs of different plant species and sesquiterpene synthases from other Asteraceae species. The AaCPS1 (GenBank accession number, AGR40502.1) was clustered in the same clade with its isomeric form of AaCPS1 (GenBank accession number, PWA38292.1). Similarly, previously characterized AaCPS (QHS1, GenBank accession number, AAL79181.1) was clustered in the same clade with AaCPS2 (GenBank accession number, PWA41248.1). The phylogenetic analysis showed a close relationship particularly among the CPSs protein sequences of *A. annua*. Also, it reveals that most of the CPSs from *Artemisia* and other plant species fall into a distinct clade of the CPSs (Fig. 2) except an uncharacterized CPS from *Chrysanthemum indicum* (GenBank accession number, AUJ87601.1). Interestingly, eight of the eleven CPSs seem to have a common origin with AaCPS3 (GenBank accession number, PWA48135.1) of *A. annua*.

### Transient expression and purification of AaCPS1 in *N. benthamiana*

A full-length cDNA of *AaCPS1* was amplified with specific primers and cloned into the pEAQ-*HT* vector in a way to generate a histidine tag (six His residues) at the C-terminal after translation. Sequence analysis revealed that the gene was inserted in the proper orientation to express the *AaCPS1* gene.

**Figure 3:**
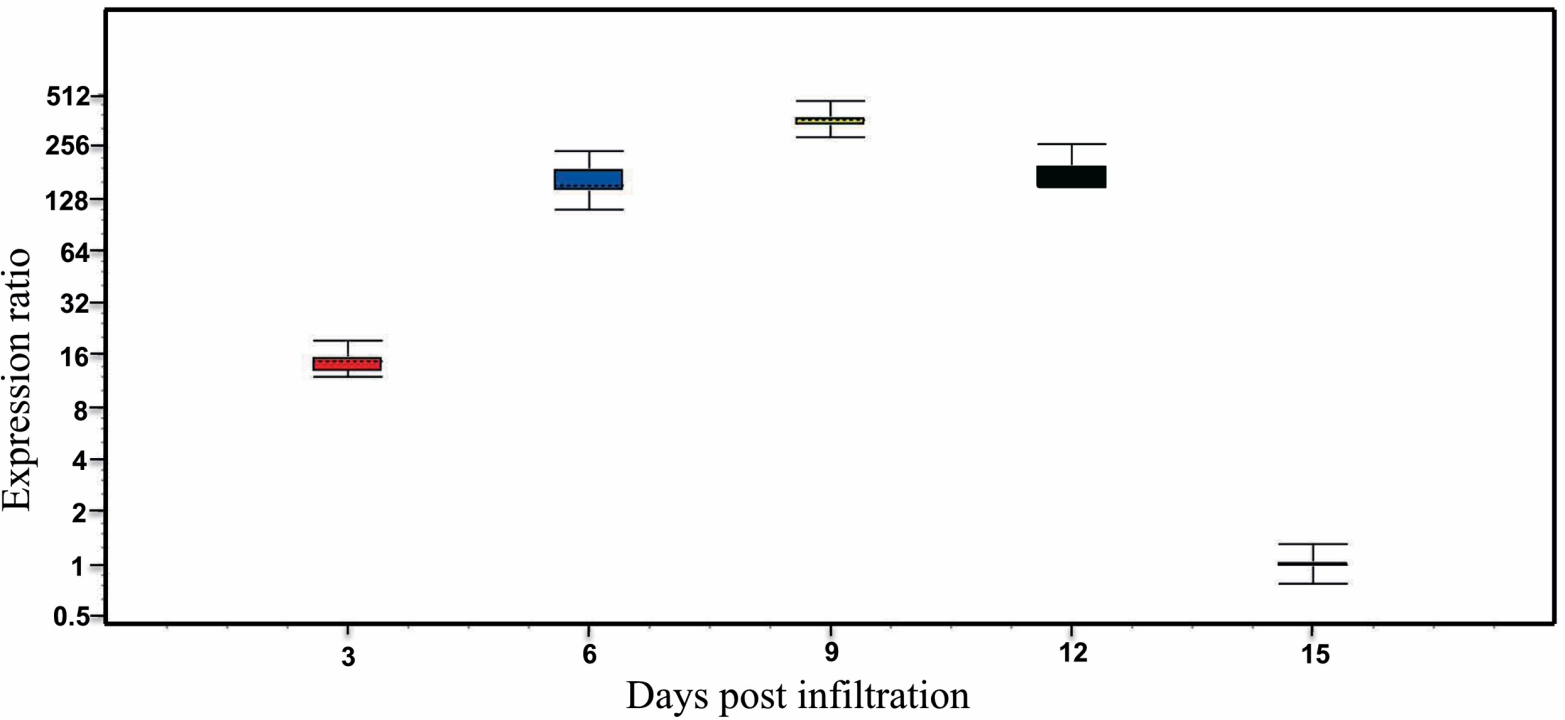
Real-time PCR analysis for β-caryophyllene synthase (*AaCPS1*) of *Artemisia annua* (*AaCPS1*) expression. Relative expression of *AaCPS1* at 3-, 6-, 9-, 12- and 15-days post infiltration (dpi) with that of 15 dpi. *ACTIN* and *elongation factor 1* (*EF1*) were used as internal controls. The error bars represent the means SD (standard deviation) from three biological and three technical replicates.

To detect and compare the gene expression levels of *AaCPS1*, the qPCR analysis was employed. Amplification efficiencies of *AaCPS1* and the reference genes, *ACTIN* and *EF1*, were found to be 89%, 94% and 91%, respectively, indicating relevant amplification and accurate quantification of the transcripts by qPCR. The homogeneity and specificity of amplified products were confirmed by melting curve analysis (Fig. S8). *AaCPS1* transcripts were clearly detected in all samples infiltrated with the vector harboring the gene of interest, but levels of expression vary at different time points of sampling. Expression levels were low on 3 days past infiltration (dpi), but increased significantly on 6 and 9 dpi. The highest level of expression was achieved on 9 dpi and the lowest level of expression was noticed on 15 dpi. The quantitative analysis of *AaCPS1* revealed the transcript level at 9 dpi was approximately 500-fold higher expression as compared to that at 15 dpi (Fig. 3).

**Figure 4:**
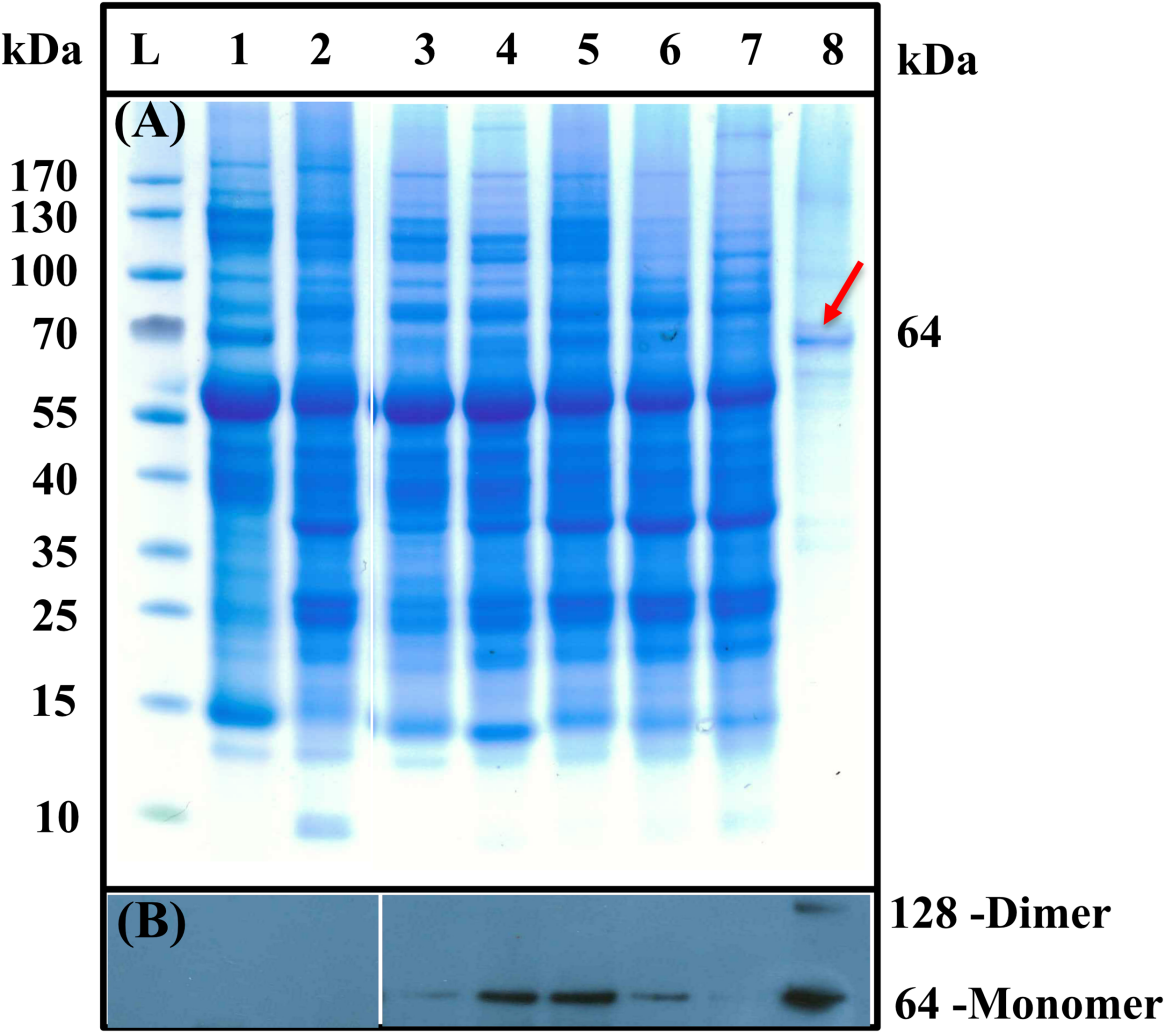
SDS–PAGE (A) and western blot (B) analysis of β-caryophyllene synthase (*AaCPS1*) expressing construct in *Nicotiana benthamiana*. Lane L, molecular weight marker; lane 1, negative control (*Nicotiana benthamiana*) lane 2, negative control (*N. benthamiana* containing an empty vector); lanes 3 to 7, total soluble proteins (TSP) were extracted from the agroinfiltrated leaves of *N. benthamiana* at 3, 6, 9, 12- and 15-days post infiltration (dpi); lane 8, purified enzyme from TSP at 9 dpi. Molecular mass of recombinant protein is indicated to the right side of the panel. Equal protein amounts (7 µg of total soluble protein or 0.5 µg of purified proteins) were loaded in each lane. No degradation fragments of the AaCSP1 were observed in the western blot.

SDS–PAGE and western blot were performed in order to verify the purification efficiency and to determine if *AaCPS1* was translated in vivo using TSP from leaves expressing the *AaCPS1* gene at different time points along with TSP from negative controls, such as the uninfiltrated leaves and the empty vector infiltrated leaves. The presence of a strong band corresponding to a molecular weight of approximately 64 kDa on the western blot revealed the translation of the *AaCPS1* transcript into the protein in infiltrated *N. benthamiana* leaves (Fig. 4). Moreover, the AaCPS1 protein was not detected in negative controls. The accumulation of AaCPS1 protein was observed in the samples on 3, 6, 9, 12 and 15 dpi while the strongest accumulation was detected at 9 dpi which is consistent to the gene expression analysis stated above (Figs. 3 and 4). The purified AaCPS1 was also confirmed by SDS–PAGE (Fig. 4). Three biological replicates were used for each time point, and three technical replicates were analyzed for each biological replicate.

Protein purification yielded 89 mg of AaCPS1 protein kg^−1^ fresh weight (FW) of leaves. Affinity purification efficiently enhanced the AaCPS1 protein purity, which is evident from the SDS–PAGE where only a few additional bands were present in the purified sample. The purity was estimated as >70%. Purification efficiency of AaCPS1 was >90 % (Fig. 4).

### Production of β-caryophyllene

**Figure 5:**
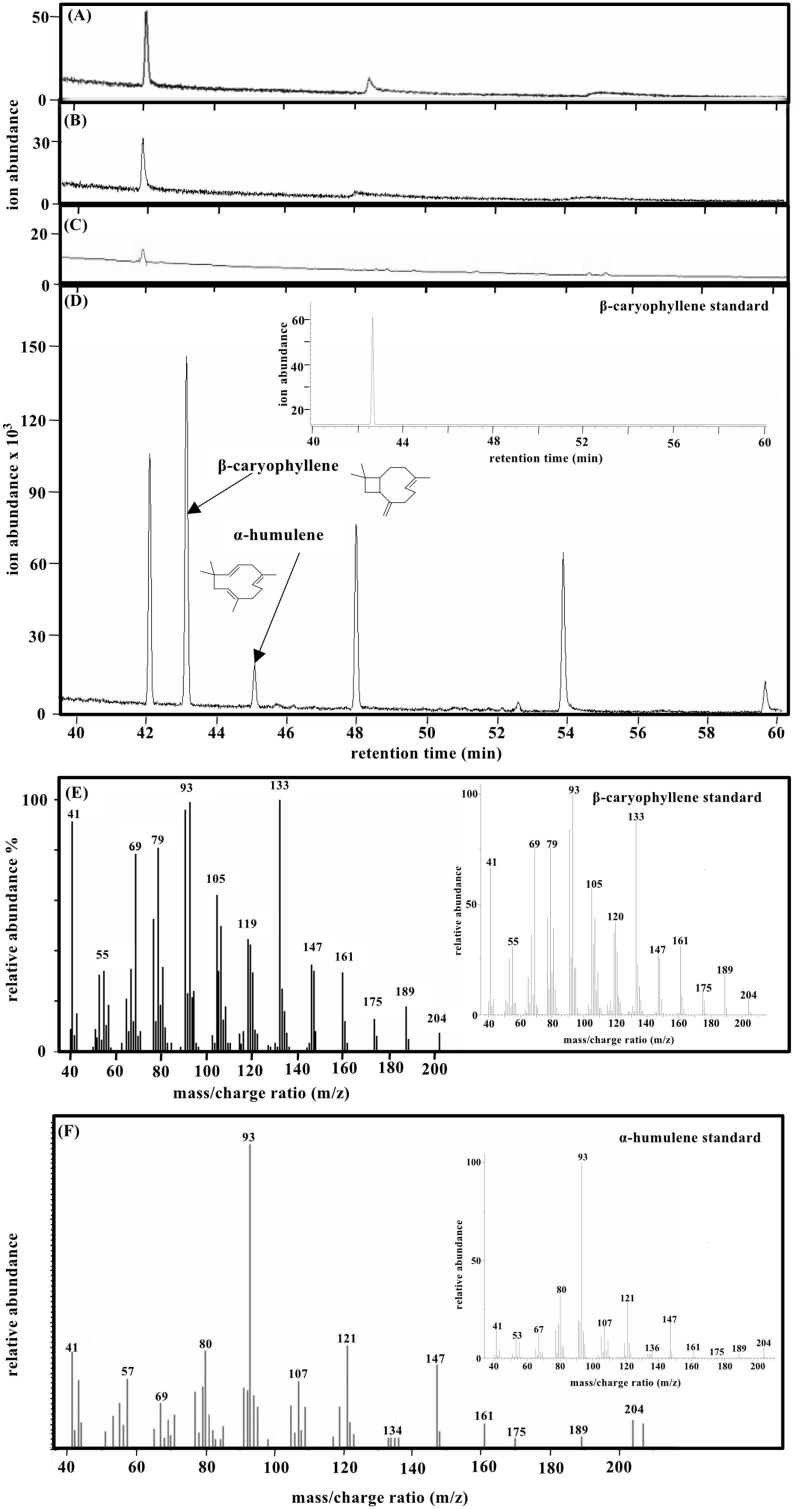
Gas chromatography–mass spectrometry (GC-MS) analysis of the formation of β-caryophyllene from farnesyl diphosphate (FPP) by recombinant β-caryophyllene synthase (AaCPS1). Gas chromatograms of the products were obtained after incubating with 20 µM FPP. (A) Total ion chromatogram (TIC) of in vitro products formed by total soluble protein from empty vector (pEAQ-*HT* and pJL3:p19) infiltrated *N. benthamiana* leaves. (B) Total ion chromatogram (TIC) of in vitro products formed by total soluble protein from *N. benthamiana* leaves. (C) Total ion chromatogram (TIC) of in vitro products formed by purified protein from empty vector (pEAQ-*HT* and pJL3:p19) infiltrated *N. benthamiana* leaves. (D) Total ion chromatogram (TIC) of in vitro products formed by the purified AaCPS1 (~10 µg) of *Artemisia annua* (AaCPS1) produced in *N. benthamiana*. The inside figure shows the total ion chromatogram (TIC) of β-caryophyllene. (E) Mass spectrum of β-caryophyllene, the inside figure shows the mass spectrum of β-caryophyllene standard. (F) Mass spectrum of α-humulene, the inside figure shows the mass spectrum of α-humulene standard.

**Figure 6:**
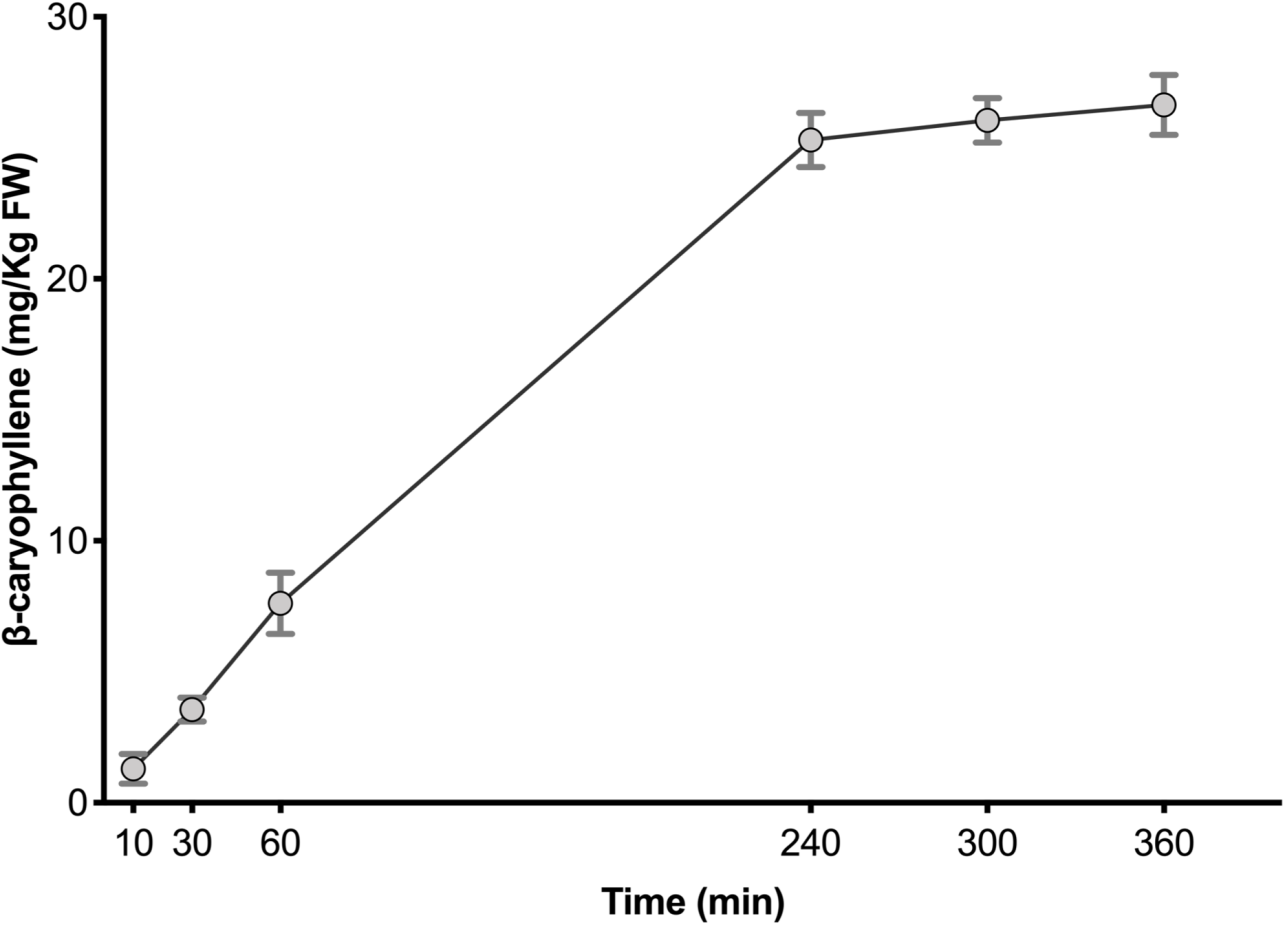
The time course production of β-caryophyllene by β-caryophyllene synthase purified from *Nicotiana benthamiana*. Error bars presented in the results indicated the standard deviation from the mean (*n* = 3).

To determine the enzyme activity and quantify AaCPS1 produced β-caryophyllene in vitro, the purified AaCPS1 was incubated with the substrate FPP. Comparison of the purified β-caryophyllene extract and TSP from the *N. benthamiana* leaves infiltrated with the empty vector at 9 dpi, TSP from the *N. benthamiana* leaves and purified extract from the *N. benthamiana* leaves infiltrated with the empty vector at 9 dpi with FPP revealed a presence of peaks in the purified AaCPS1 extract at the retention time of 42.74 min and 44.57 min (Figs. 5A–5D). These peaks were confirmed to be β-caryophyllene and α-humulene by comparison of their mass spectra to that of standard β-caryophyllene and α-humulene (Figs. 5E and 5F). Under the assay conditions used in this study, we were able to obtain a maximum production 27.5 mg of β-caryophyllene kg^−1^ FW in 6 h and the AaCPS1 enzyme was active for 240 min in a linear manner (Fig. 6). These results suggested a great potential for using the *N. benthamiana* expression systems to produce CPSs which could then be used for producing β-caryophyllene on a large scale. Three biological replicates were used to quantify the β-caryophyllene produced by AaCPS1.

## Discussion

Due to the wide applications of β-caryophyllene in food and medicine, the possibility of its large scale and more sustainable production has received much attention in recent years. The sesquiterpene β-caryophyllene, is a ubiquitous compound that is widely distributed and produced by many members of the plant kingdom. Though several full-length putative *CPS* genes have been identified so far in *A. annua*, only one CPS protein (GenBank accession number, AAL79181.1) has been functionally characterized or produced up to date, to our knowledge (Cai et al., 2002).

In order to produce β-caryophyllene in vitro, the full-length isoform of *AaCPS1* gene identified in this study was used. The identified gene, *AaCPS1* encodes a predicted polypeptide of 550 amino acid residues, with a predicted molecular mass of 63.99 kDa corresponding to the class of terpene synthases in plants. In general, terpene synthases (TPSs) have 550–850 amino acids with molecular mass ranging between 50 and 100 kDa. Sesquiterpene synthases are usually localized in the cytosol where FPP serves as the substrate (Danner et al., 2011) and no signal peptide was predicted for AaCPS1, suggesting that this enzyme function as sesquiterpene synthase in the cytosol. As β-caryophyllene biosynthesis takes place in the cytosol, which lacks a plastid targeting signal and is typically shorter than mono- and diterpene synthases. Comparison of *AaCPS1* and their high sequence identity to that of PKPP01015792.1 at the nucleotide and amino acid level (>99%, Figs. S1 and S5) revealed these two *CPS* paralogs to be two alleles rather than different genes.

The *CPS* genes have evolved over time, but they still show high DNA sequence homology. For instance, the sequence of *AaCPS1* clearly resembles (M80% identity) the *CPS*s of other plants. The comparison revealed the presence of several highly conserved regions of this enzyme, which is required for binding of the substrate (FPP) of these enzymes. At the C-terminal part of the protein, the highly conserved RDR, and aspartate rich (DDxxD) motifs, and less conserved aspartate rich (NSE/DTE) motif are present. The DDxxD (DDTYD) and NSE/DTE (LMDDIHSQKEE) motifs have been reported to flank the entrance of the active site and are involved in divalent metal ion binding, which is essential for enzyme-substrate binding and catalytic function (López-Gallego, Wawrzyn & Schmidt-Dannert, 2010). The conserved motifs of RXR, DDxxD and NSE/DTE are important for sesquiterpene synthase functionality in the complexing of the diphosphate group, after the ionization of FPP (Starks et al., 1997). The DDxxD motif binds two divalent cations (Mg^2+^) and the NSE/DTE motif binds one magnesium ion forming a tri-nuclear divalent metal ion cluster, which is involved in the binding of the diphosphate of the allylic substrate, FPP (Christianson, 2006). The NSE/DTE motif sequence is conserved in all the AaCPSs except for the insert of 35 amino acids in AaCPS3. This unique insert may be a genome sequencing artifact. The sequence comparison among the CPSs indicates that the AaCPS1 is structurally similar to other plant sesquiterpene synthases.

Comparison of the protein sequences of the *Artemisia* CPSs with other CPSs indicated putative evolutionary origin of sesquiterpene synthases from β-farnesene synthase (Genbank accession number, AAX39387.1). Based on the phylogenetic analysis using protein sequences, AaCPS1 clustered on the same group with seven other CPSs. As expected, all putative enzyme isoforms and full length CPSs of *A. annua* claded together and subdivided in two subfamilies (Fig. 2).

The plant virus-based vector, pEAQ-*HT*-AaCPS1 along with the viral suppressor (P19), was transformed into *Agrobacterium* strain, LBA4404 to achieve a higher amount of recombinant protein production. Previous studies also demonstrated that *Agrobacterium* strain, LBA4404 had higher expression potential than other strains of *Agrobacterium* in *N. benthamiana* (Kanagarajan et al., 2012a). The detection of transcript and protein accumulation patterns of the *AaCPS1* in *N. benthamiana* showed gradual increase of transcription until 9 dpi. These results suggest that *AaCPS1* mRNA started to degrade after 9 dpi, which lead to decline in transcript abundance at 12 dpi and 15 dpi. The results are consistent with previous studies of transient gene expression using viral vector in *N. benthamiana* (Ye et al., 2011; Kanagarajan et al., 2012a). We can hypothesize that accumulation of CPMV proteins in agroinfiltrated leaves of *N. benthamiana* may cause leaf necrosis in the infiltrated areas at 12 and 15 dpi. However, these results contradict with previous studies demonstrating that transient green florescent protein expression was remained at the same expression level up to 15 dpi (Lindbo, 2007). This higher level of expression in leaves reported in the current study indicates a high capacity to produce recombinant enzymes in *N. benthamiana* by transient gene expression using tobacco mosaic virus-based vector. Similar trend of protein accumulation was observed in the western blot. This is expected as most of the sesquiterpene synthases are unstable over time and Protparam analysis of AaCPS1 also showed instability index of AaCPS1 as 46.51, which classifies AaCPS1 as an unstable protein. Also, the estimated purification efficiency and purity of AaCPS1 were in line with previous studies using tobacco leaves as expression platform for producing recombinant proteins (Bendandi et al., 2010; Pogue et al., 2010).

The protein, AaCPS1 used in this work could produce β-caryophyllene as a main compound and α-humulene as a second product. Both the sesquiterpene products may be produced by a common reaction pathway (Fig. S9). CPS produced in *E. coli* has also been reported to synthesize both β-caryophyllene and α-humulene from FPP (Cai et al., 2002). There are three unknown byproducts which were found in the negative controls (Figs. 5A and 5B). None of the three byproducts exhibits typical molecular ion peaks of sesquiterpenes, that is, 204 or 222 for aliphatic and hydroxylated sesquiterpene, respectively. FPP is an intermediate product of the sesquiterpene biosynthetic pathway in many plants, including *N. benthamiana*. However, *N. benthamiana* may not produce sufficient amounts of FPP for the production of any significant amounts of β-caryophyllene although AaCPS1 is available in vivo as most of the native FPP is used to produce endogenous sesquiterpenes and other products. During our enzyme assay, our protein samples were overlaid with dodecane, because β-caryophyllene is a volatile sesquiterpene, which reacts readily with ozone (O3) and other reactive oxygen species (ROS) (Grosjean et al., 1993). In addition, continuous extraction of the lipophilic product may improve the yield by pulling the product from the reaction mixture preventing product inhibition. Furthermore, the *k*_cat_ of terpene synthases is very low (*k*_cat_ < 0.1 s^−1^) due to the fact that the release of product is rate limiting for the enzymatic reaction due to low solubility of the lipophilic product. By continuous extraction of the product an improvement of the *k*_cat_ may be expected, which may be of importance for large scale production of β-caryophyllene.

In our assay, the formation of β-caryophyllene was linear for 4 h indicating that the recombinant enzyme was stable under the condition used (Fig. 6). After 4 h, the amount of product obtained corresponded to a complete conversion of the FPP (10 nmoles) added to the assay. These results show that volatile sesquiterpene, β-caryophyllene can be quantitatively produced by a recombinant enzyme obtained from *N. benthamiana* transient expression system.

Our main objective of this study is to establish that our isolated duplicated gene of AaCPS1 is functional and can be used for further applications or studies. There are several sesquiterpene synthases, for example, *epi*-cedrol synthase (ECS), amorpha-4,11-diene synthase (ADS), β-caryophyllene synthase (CPS), (E)-β-farnesene synthase (FS) and germacrene A synthase (GS) as well as squalene synthase compete for FPP in *A. annua*. However, ADS is the only enzyme involved in artemisinin biosynthesis. Numerous studies have been carried out to block the expression of sesquiterpene synthase(s) competing for FPP by antisense technology or RNA interference to enhance the biosynthesis of artemisinin (Chen et al., 2011; Wang et al., 2012; Lv et al., 2016). But, none of the studies have been carried out with all of the isoforms of CPS. In the future, outcome of the present study will help us to downregulate/inhibit competitive sesquiterpene synthase(s), which may redirect the metabolic flux resulting in an increase in artemisinin production.

Microbial production systems, such as engineered yeast and bacteria, have also been demonstrated the potential to meet the demand for β-caryophyllene. However, the major drawback in the production of CPS using microbial expression systems is the formation of inclusion bodies. It emphasizes the need to explore other host systems that have a native protein synthesis. Recent studies on the transient heterologous expression in *N. benthamiana* reflects the growing popularity of plant-based expression platform for the production of terpenoids (Ting et al., 2015; Wang et al., 2016; Reed et al., 2017). However, the present high cost of the substrate will make such a production system unrealistic. The way to go may be the production of a fusion of FDS and CPS in line with our previous studies on the fusion of FDS and ADS (Han et al., 2016) and on the fusion of FDS and tobacco *epi*-aristolochene synthase (Brodelius et al., 2002). Such a fusion protein would use IPP and DMAPP as substrates. These substrates can be produced chemically in good yields from cheap precursors. In addition, the fused protein may be immobilized on a solid support opening for a continuous production of β-caryophyllene with external extraction. More recently, simple spraying of *N. benthamiana* plants with *Agrobacterium* vectors was found to be suitable for cost-effective and large-scale production of pharmaceutically and industrially valuable proteins (Hahn et al., 2015; Schulz et al., 2015; Carlsson et al., 2020).

## Conclusion

In this study, we successfully produced β-caryophyllene using an enzyme, AaCPS1 synthesized in *N. benthamiana*. The ability to produce CPS in *N. benthamiana* using transient expression is a promising alternative to microorganisms as it produces soluble CPS that can efficiently synthesize β-caryophyllene without genetic modification, which is of relevance to the plant and therapeutic research communities. In future, more robust transient plant expression of plant proteins has the potential to be a more efficient platform for the production of important terpenoids.

## Supplemental Information

10.7717/peerj.8904/supp-1 Supplemental Information 1 Nucleotide sequence alignment of β-caryophyllene synthase (*AaCPS1*) gene of *Artemisia annua* with previously characterized *CPS* gene (*QHS1 or AaCPS2*) of *A. annua*. GenBank accession number of *AaCPS1*, KC118534.1. GenBank accession number *QHS1 or AaCPS2*, AF472361.1. Nucleotide differences of the *AaCPS* genes are shown with an asterisk (*).

10.7717/peerj.8904/supp-2 Supplemental Information 2 Nucleotide sequence alignment of β-caryophyllene synthase (*AaCPS1*) isoforms (GenBank accession number, KC118534.1 and GenBank accession number, PKPP01015792.1) of *Artemisia annua*. Sequence alignment showing the single base differences (9th, 115th, 395th, 1,101th and 1,515th position) of *AaCPS1* isoforms in the coding sequences. Nucleotide differences of the *AaCPS* genes are shown with an asterisk (*).

10.7717/peerj.8904/supp-3 Supplemental Information 3 Nucleotide sequence alignment of β-caryophyllene synthase (*AaCPS1*) gene of *Artemisia annua* with recently reported *AaCPS* of *A. annua*, *AaCPS2*. GenBank accession number of *AaCPS1*, KC118534.1. GenBank accession number *AaCPS2*, PKPP01013206.1. Nucleotide differences of the *AaCPS* genes are shown with an asterisk (*).

10.7717/peerj.8904/supp-4 Supplemental Information 4 Nucleotide sequence alignment of β-caryophyllene synthase (*AaCPS1*) gene of *Artemisia annua* with recently reported *AaCPS* of *A. annua*, *AaCPS3*. GenBank accession number of *AaCPS1*, KC118534.1. GenBank accession number of *AaCPS3*, PKPP01009489.1. Nucleotide differences of the *AaCPS* genes are shown with an asterisk (*).

10.7717/peerj.8904/supp-5 Supplemental Information 5 Alignment of the deduced amino acid sequences of β-caryophyllene synthase (*AaCPS1*) gene with its isomeric form of *AaCPS1* gene of *Artemisia annua*. GenBank accession number of *AaCPS1* AGR40502.1. GenBank accession number of its isomeric form, PWA38292.1. Boxes: conserved amino acid sequence motifs of sesquiterpene synthases (RxR, DDxxD and NSE/DTE). Circles: amino acid differences between the two enzyme isoforms. No signal peptide was predicted in the N-terminus of the AaCPS1 protein.

10.7717/peerj.8904/supp-6 Supplemental Information 6 Alignment of the deduced amino acid sequences of β-caryophyllene synthase gene, *AaCPS1* with three other AaCPS proteins, AaCPS1, AaCPS2 and AaCPS3 of *Artemisia annua*. GenBank accession number of AaCPS1, AGR40502.1. GenBank accession number of AaCPS1 PWA38292.1. GenBank accession number of AaCPS2, PWA41248.1. GenBank accession number, AaCPS3, PWA48135.1. Boxes: conserved amino acid sequence motifs of sesquiterpene synthases (RxR, DDxxD and NSE/DTE). Amino acid differences of the AaCPS proteins are shown with an asterisk (*).

10.7717/peerj.8904/supp-7 Supplemental Information 7 Alignment of the deduced amino acid sequences of β-caryophyllene synthase gene (*AaCPS1*) with sesquiterpene synthases of Asteracean species. *Artemisia annua*, caryophyllene synthase, AaCPS1 (GenBank accession number, AGR40502.1); *A. annua*, caryophyllene synthase, AaCPS1 (GenBank accession number, PWA38292.1); *A. annua*, beta-caryophyllene synthase, AaCPS2 (GenBank accession number, PWA41248.1), *A. annua*, E-beta-caryophyllene synthase, AaCPS3 (GenBank accession number, PWA48135.1), *A. absinthium*, beta-caryophyllene synthase (GenBank accession number, BAN81914.1), *Tanacetum parthenium*, E-beta-caryophyllene synthase (GenBank accession number, AEH41845.1), *Achillea millefolium*, beta-caryophyllene synthase (GenBank accession number, AGZ84810.1), *Matricaria chamomilla* var. *Recutita*, beta-caryophyllene synthase (GenBank accession number, I6RAQ6.1), *Lactuca sativa*, beta-caryophyllene synthase-like (GenBank accession number, XP_023750885.1), *Helianthus annuus*, beta-caryophyllene synthase-like (GenBank accession number, XP_021988936.1), *Cynara cardunculus* var. *scolymus*, beta-caryophyllene synthase-like (GenBank accession number, XP_024981250.1), *Chrysanthemum indicum*, beta-caryophyllene synthase (GenBank accession number, AUJ87601.1), *A. annua*, amorpha-4,11-diene synthase (GenBank accession number, AAF61439.1), *A. annua*, *epi*-cedrol synthase (GenBank accession number, CAC08805.1), *A. annua*, (−)-germacrene D synthase (GenBank accession number, PWA68009.1), *A. annua*, alpha-isocomene synthase (GenBank accession number, PWA85509.1), *A. annua*, alpha-bisabolol synthase (GenBank accession number, AFV40969.1), and *A. annua*, (E)-beta-farnesene synthase (GenBank accession number, AAX39387.1). Boxes: conserved amino acid sequence motifs of sesquiterpene synthases (RxR, DDxxD and NSE/DTE).

10.7717/peerj.8904/supp-8 Supplemental Information 8 Fig. S8. Melting curve analysis from a real time PCR assay. Melting curves obtained by real-time PCR amplification targeting *AaCPS1*, *Actin* and *EF1* genes

10.7717/peerj.8904/supp-9 Supplemental Information 9 Fig. S9. Reaction pathway of farnesyl diphosphate (FPP).

10.7717/peerj.8904/supp-10 Supplemental Information 10 Raw data for relative expression of *AaCPS1* at different time points, qPCR data analysis, full length images of SDS-PAGE and Western blot and time course production of β-caryophyllene.

## Additional Information and Declarations

### DNA Deposition

The following information was supplied regarding the deposition of DNA sequences: The cDNA sequence is available at GenBank: KC118534.1.

### Data Availability

The following information was supplied regarding data availability: Raw data for relative expression of AaCPS1 at different time points, qPCR data analysis, full length images of SDS–PAGE and western blot, GC–MS analysis of α-humulene, β-caryophyllene and time course production of β-caryophyllene are available in the Supplemental Files.
