# Supplementary figures and images for: Transient expression and purification of β-caryophyllene synthase in Nicotiana benthamiana to produce β-caryophyllene in vitro
Source: PeerJ. 2020 Apr 28;8:e8904. doi: 10.7717/peerj.8904 (PMC7194099; doi:10.7717/peerj.8904)

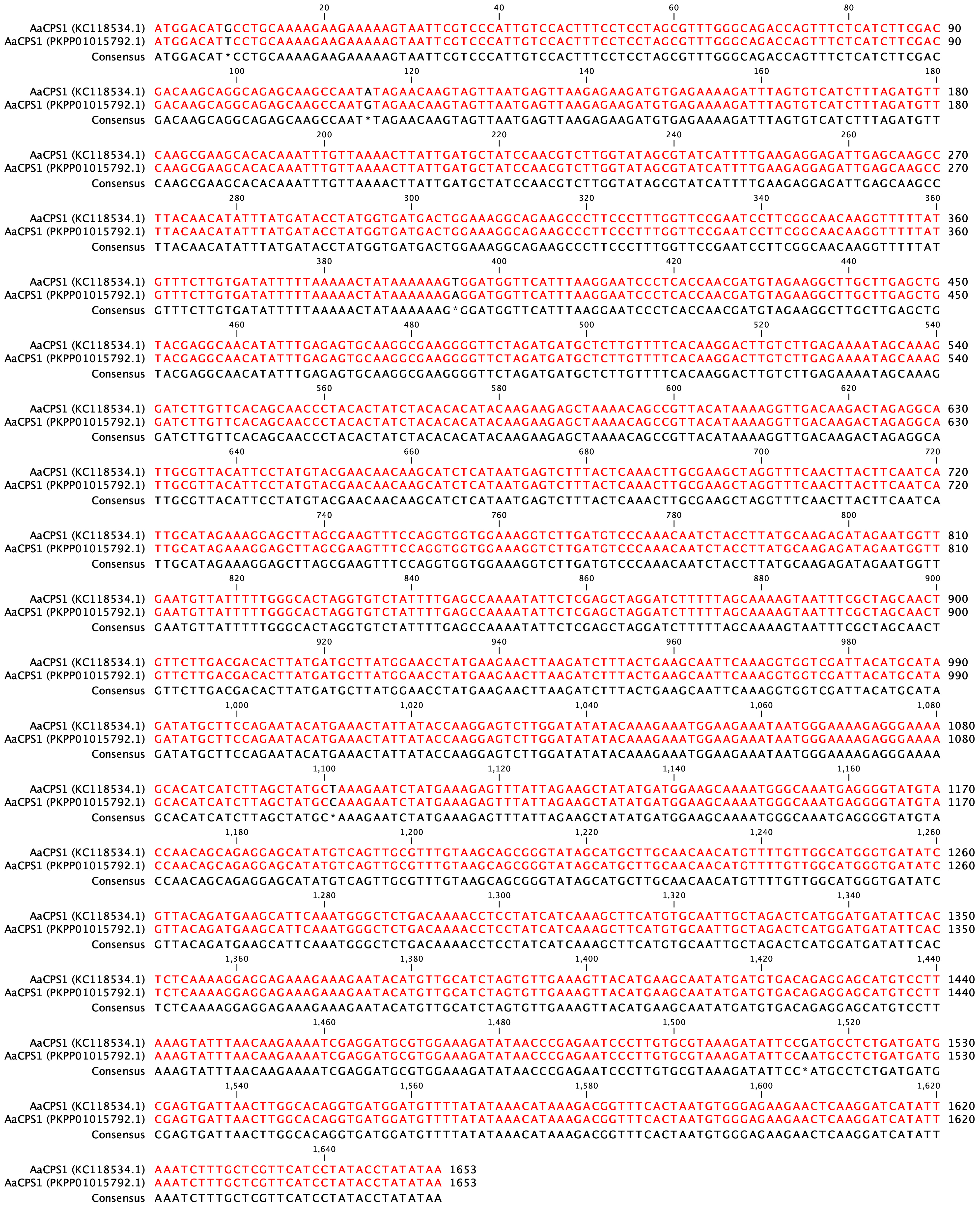

Supplement: Supplemental Information 2 — Sequence alignment showing the single base differences (9th, 115th, 395th, 1,101th and 1,515th position) of AaCPS1 isoforms in the coding sequences. Nucleotide differences of the AaCPS genes are shown with an asterisk (*). [file peerj-08-8904-s002.jpg]

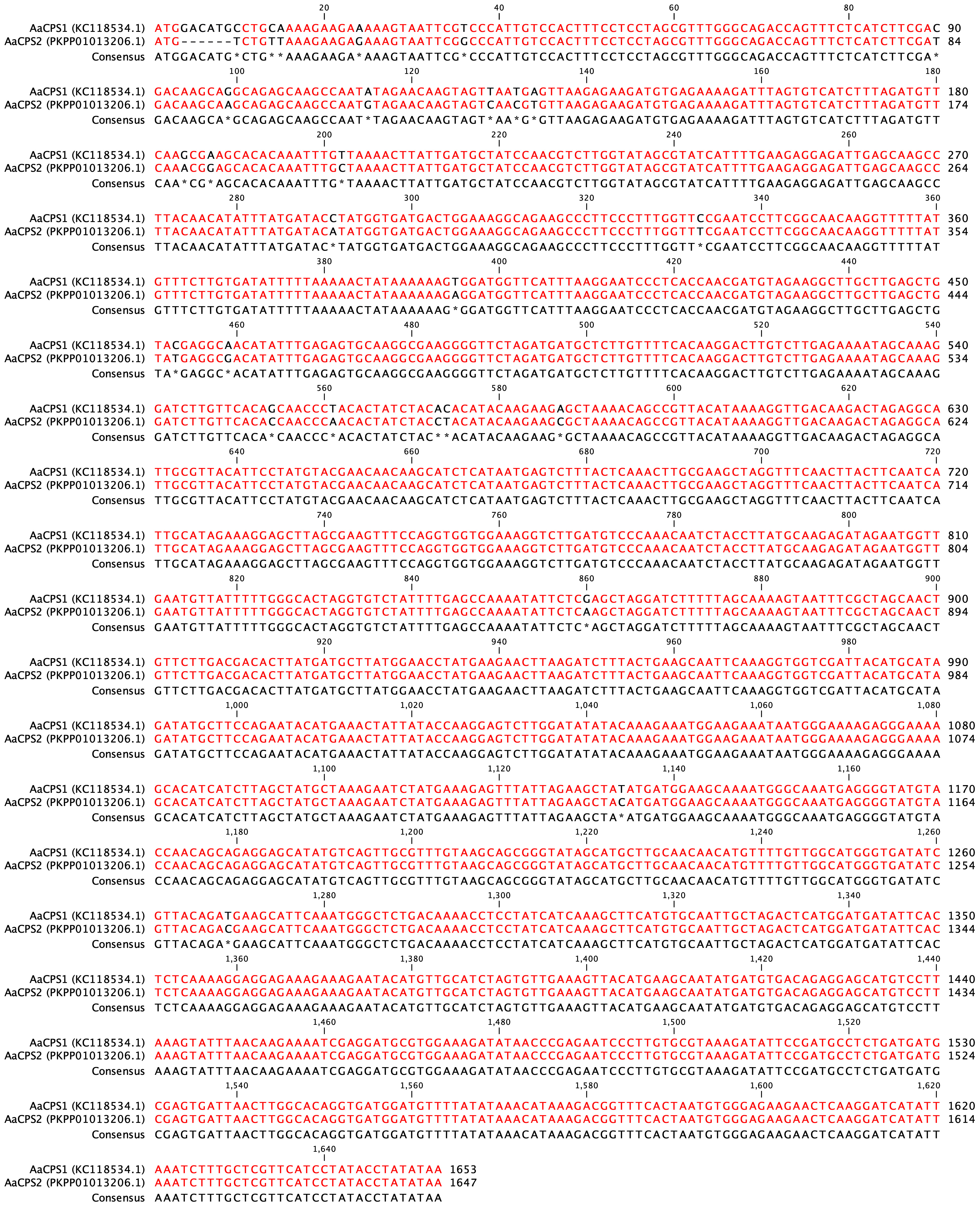

Supplement: Supplemental Information 3 — GenBank accession number of AaCPS1, KC118534.1. GenBank accession number AaCPS2, PKPP01013206.1. Nucleotide differences of the AaCPS genes are shown with an asterisk (*). [file peerj-08-8904-s003.jpg]

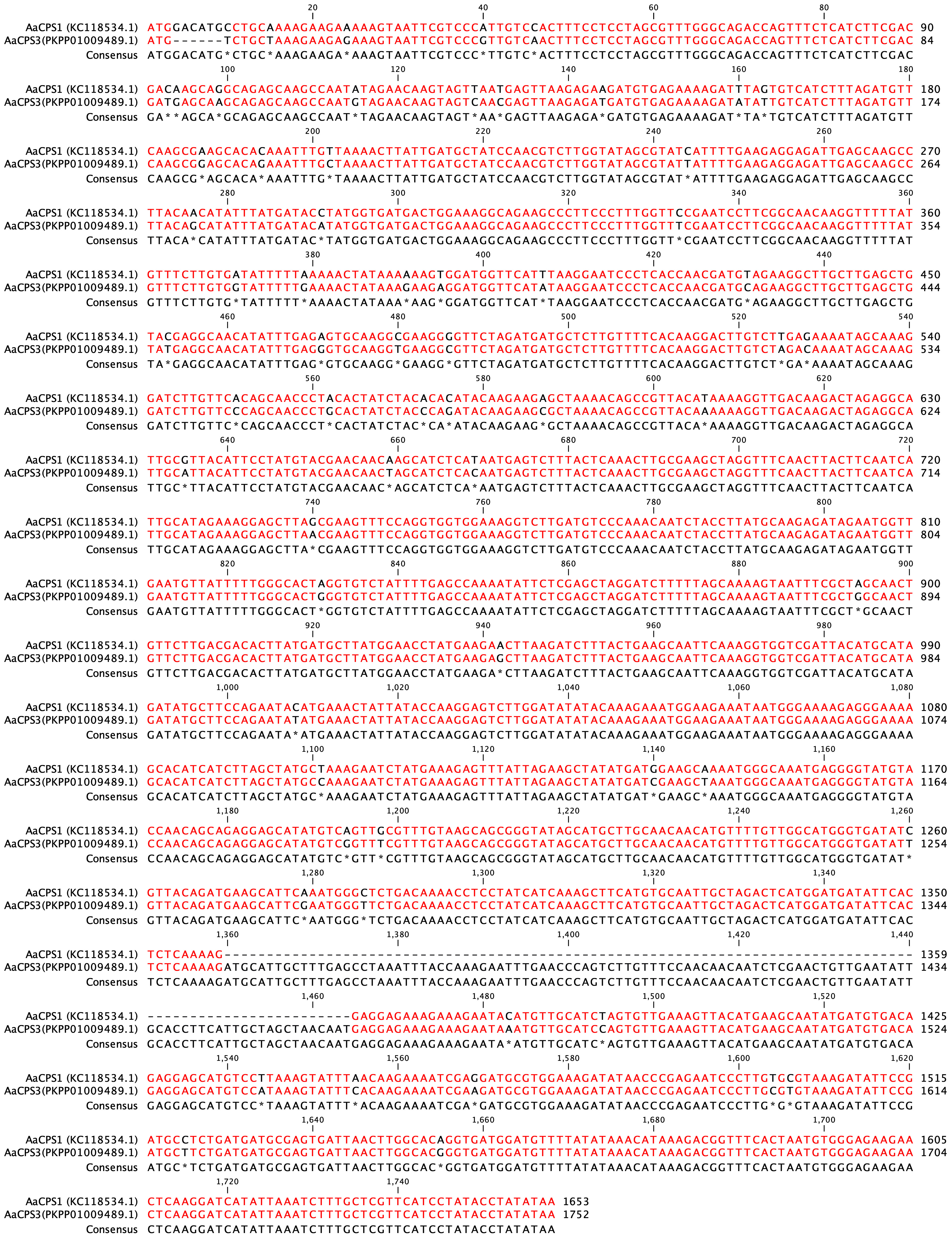

Supplement: Supplemental Information 4 — GenBank accession number of AaCPS1, KC118534.1. GenBank accession number of AaCPS3, PKPP01009489.1. Nucleotide differences of the AaCPS genes are shown with an asterisk (*). [file peerj-08-8904-s004.jpg]

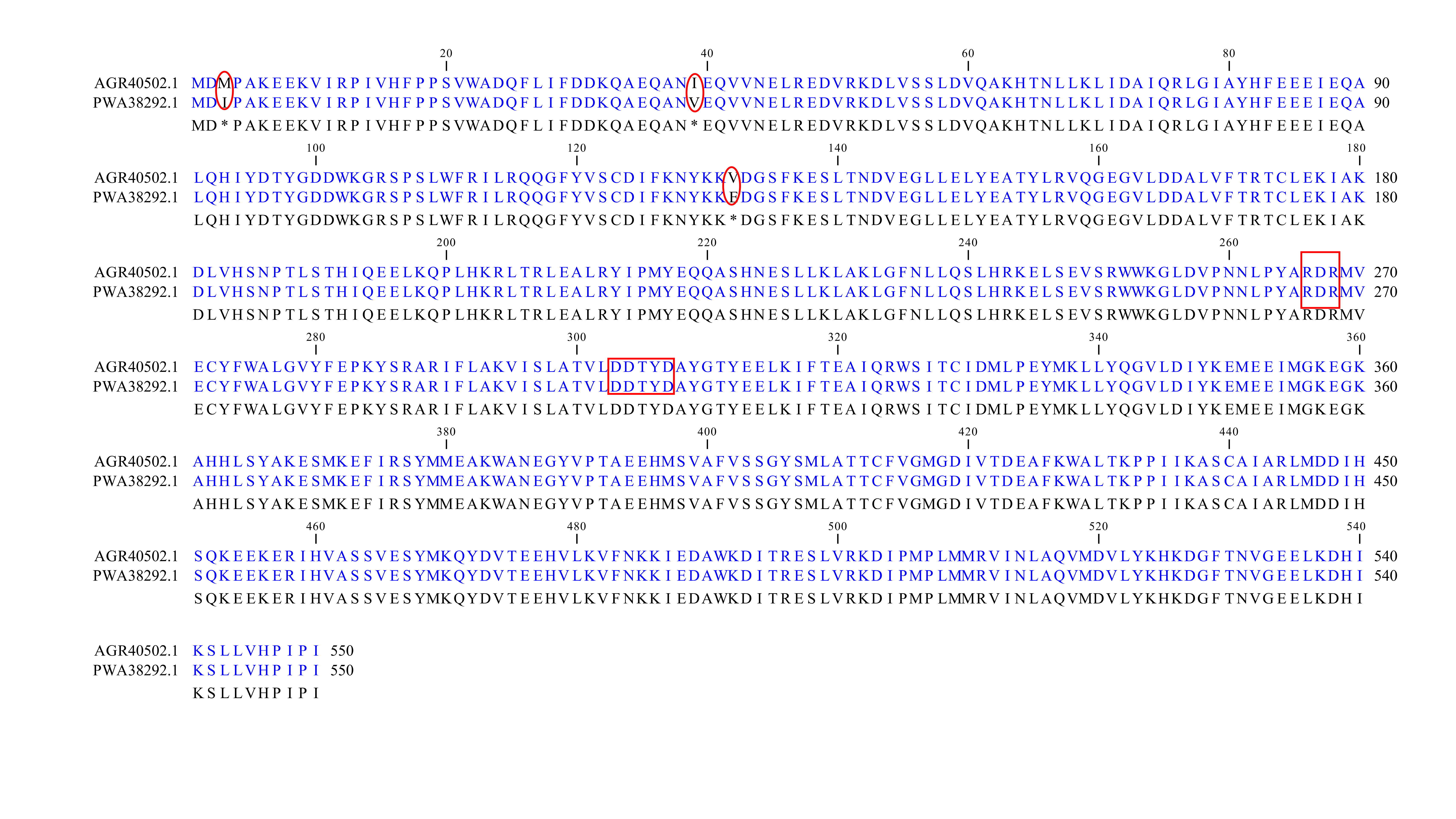

Supplement: Supplemental Information 5 — GenBank accession number of AaCPS1 AGR40502.1. GenBank accession number of its isomeric form, PWA38292.1. Boxes: conserved amino acid sequence motifs of sesquiterpene synthases (RxR, DDxxD and NSE/DTE). Circles: amino acid differences between the two enzyme isoforms. No signal peptide was predicted in the N-terminus of the AaCPS1 protein. [file peerj-08-8904-s005.jpg]

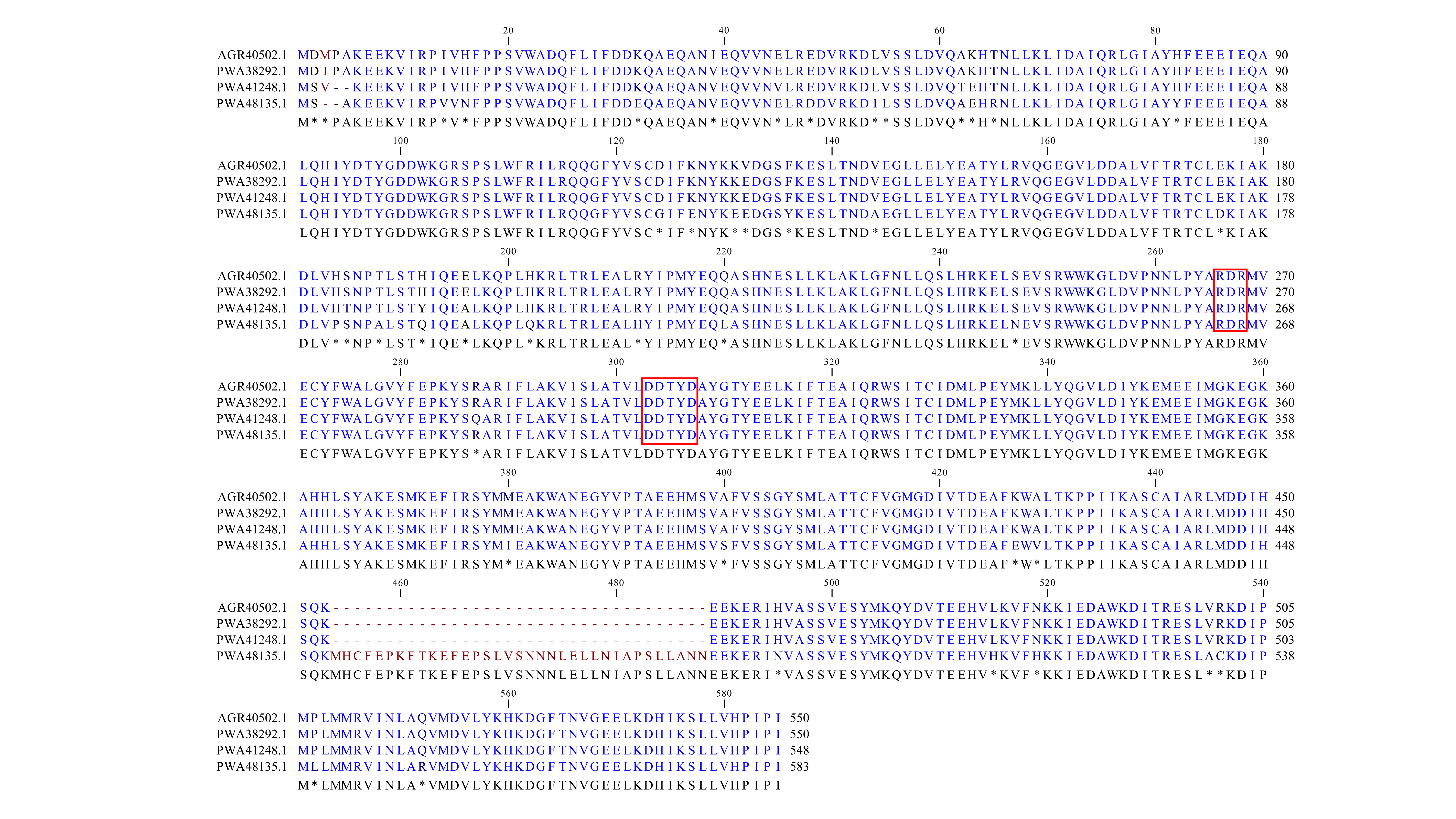

Supplement: Supplemental Information 6 — GenBank accession number of AaCPS1, AGR40502.1. GenBank accession number of AaCPS1 PWA38292.1. GenBank accession number of AaCPS2, PWA41248.1. GenBank accession number, AaCPS3, PWA48135.1. Boxes: conserved amino acid sequence motifs of sesquiterpene synthases (RxR, DDxxD and NSE/DTE). Amino acid differences of the AaCPS proteins are shown with an asterisk (*). [file peerj-08-8904-s006.jpg]

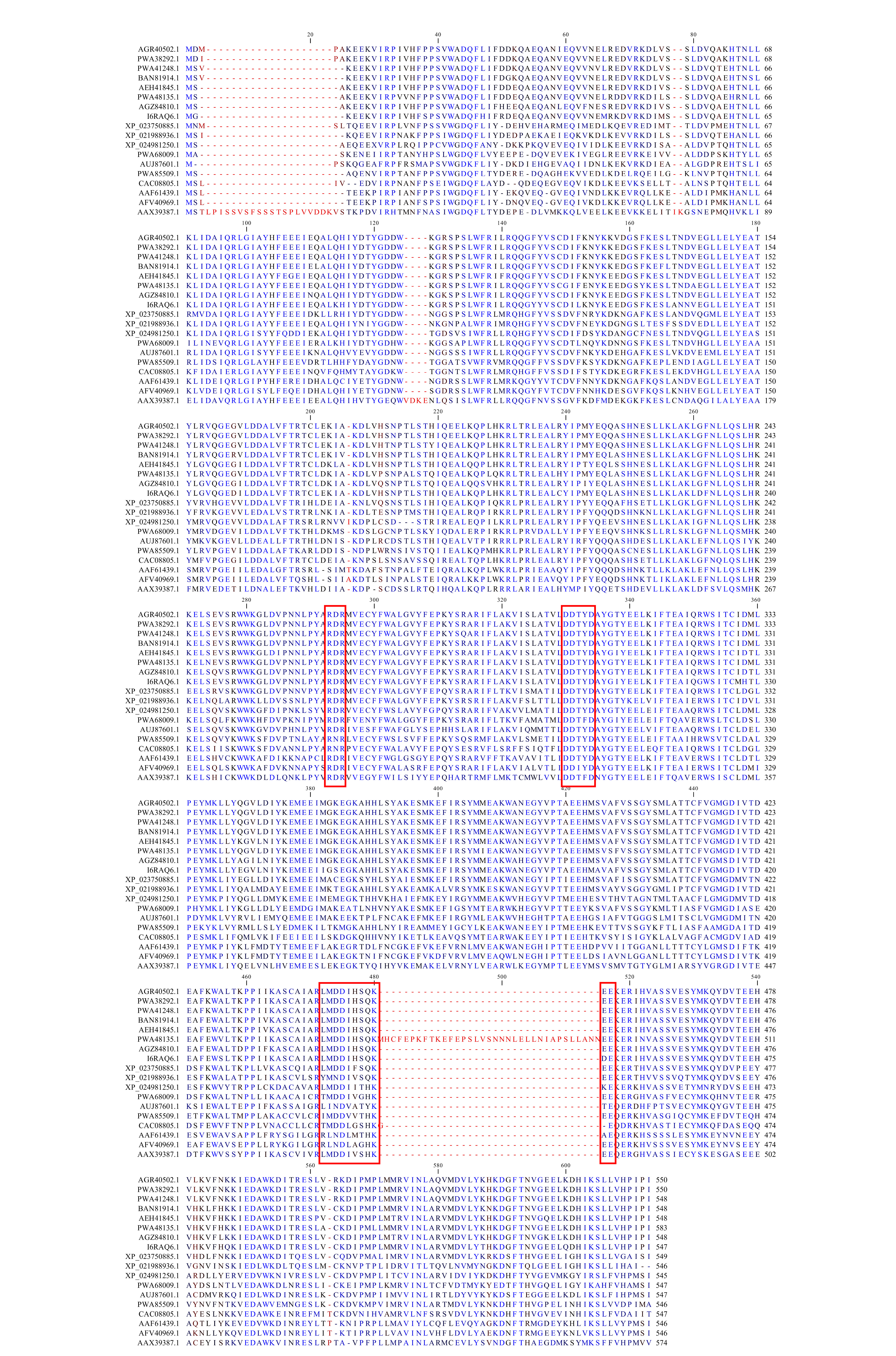

Supplement: Supplemental Information 7 — Artemisia annua, caryophyllene synthase, AaCPS1 (GenBank accession number, AGR40502.1); A. annua, caryophyllene synthase, AaCPS1 (GenBank accession number, PWA38292.1); A. annua, beta-caryophyllene synthase, AaCPS2 (GenBank accession number, PWA41248.1), A. annua, E-beta-caryophyllene synthase, AaCPS3 (GenBank accession number, PWA48135.1), A. absinthium, beta-caryophyllene synthase (GenBank accession number, BAN81914.1), Tanacetum parthenium, E-beta-caryophyllene synthase (GenBank accession number, AEH41845.1), Achillea millefolium, beta-caryophyllene synthase (GenBank accession number, AGZ84810.1), Matricaria chamomilla var. Recutita, beta-caryophyllene synthase (GenBank accession number, I6RAQ6.1), Lactuca sativa, beta-caryophyllene synthase-like (GenBank accession number, XP_023750885.1), Helianthus annuus, beta-caryophyllene synthase-like (GenBank accession number, XP_021988936.1), Cynara cardunculus var. scolymus, beta-caryophyllene synthase-like (GenBank accession number, XP_024981250.1), Chrysanthemum indicum, beta-caryophyllene synthase (GenBank accession number, AUJ87601.1), A. annua, amorpha-4,11-diene synthase (GenBank accession number, AAF61439.1), A. annua, epi-cedrol synthase (GenBank accession number, CAC08805.1), A. annua, (−)-germacrene D synthase (GenBank accession number, PWA68009.1), A. annua, alpha-isocomene synthase (GenBank accession number, PWA85509.1), A. annua, alpha-bisabolol synthase (GenBank accession number, AFV40969.1), and A. annua, (E)-beta-farnesene synthase (GenBank accession number, AAX39387.1). Boxes: conserved amino acid sequence motifs of sesquiterpene synthases (RxR, DDxxD and NSE/DTE). [file peerj-08-8904-s007.jpg]

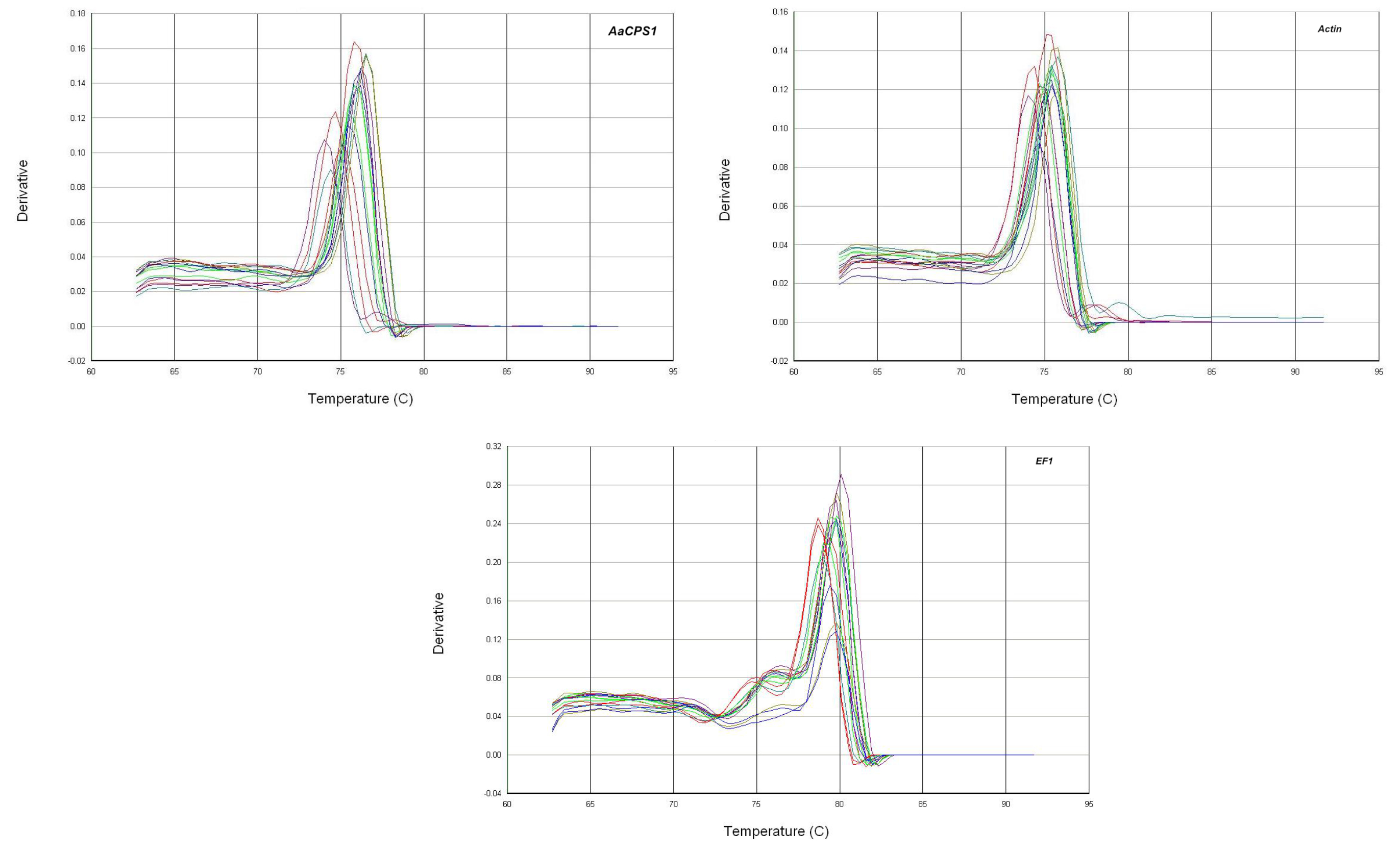

Supplement: Supplemental Information 8 — Melting curves obtained by real-time PCR amplification targeting AaCPS1, Actin and EF1 genes [file peerj-08-8904-s008.jpg]

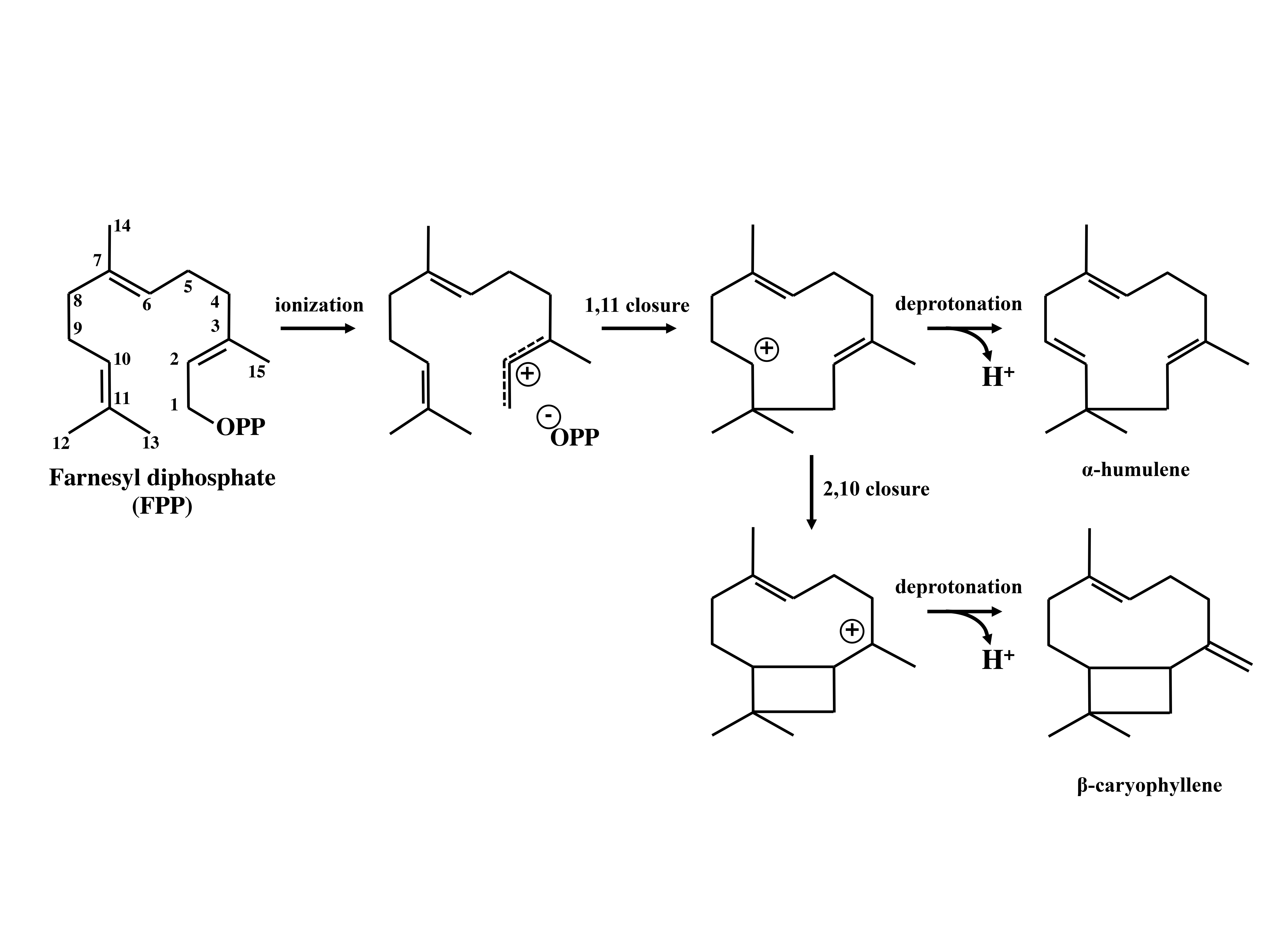

Supplement: Supplemental Information 9 [file peerj-08-8904-s009.jpg]

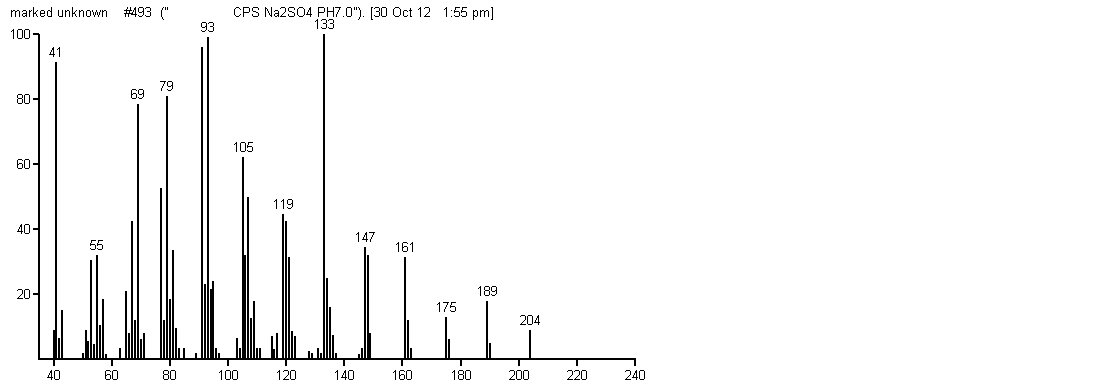

Supplement: Supplemental Information 10 [file peerj-08-8904-s010.zip › Raw data-4 Feb/Fig. 5E. CPS-1 Massspectrum.png]

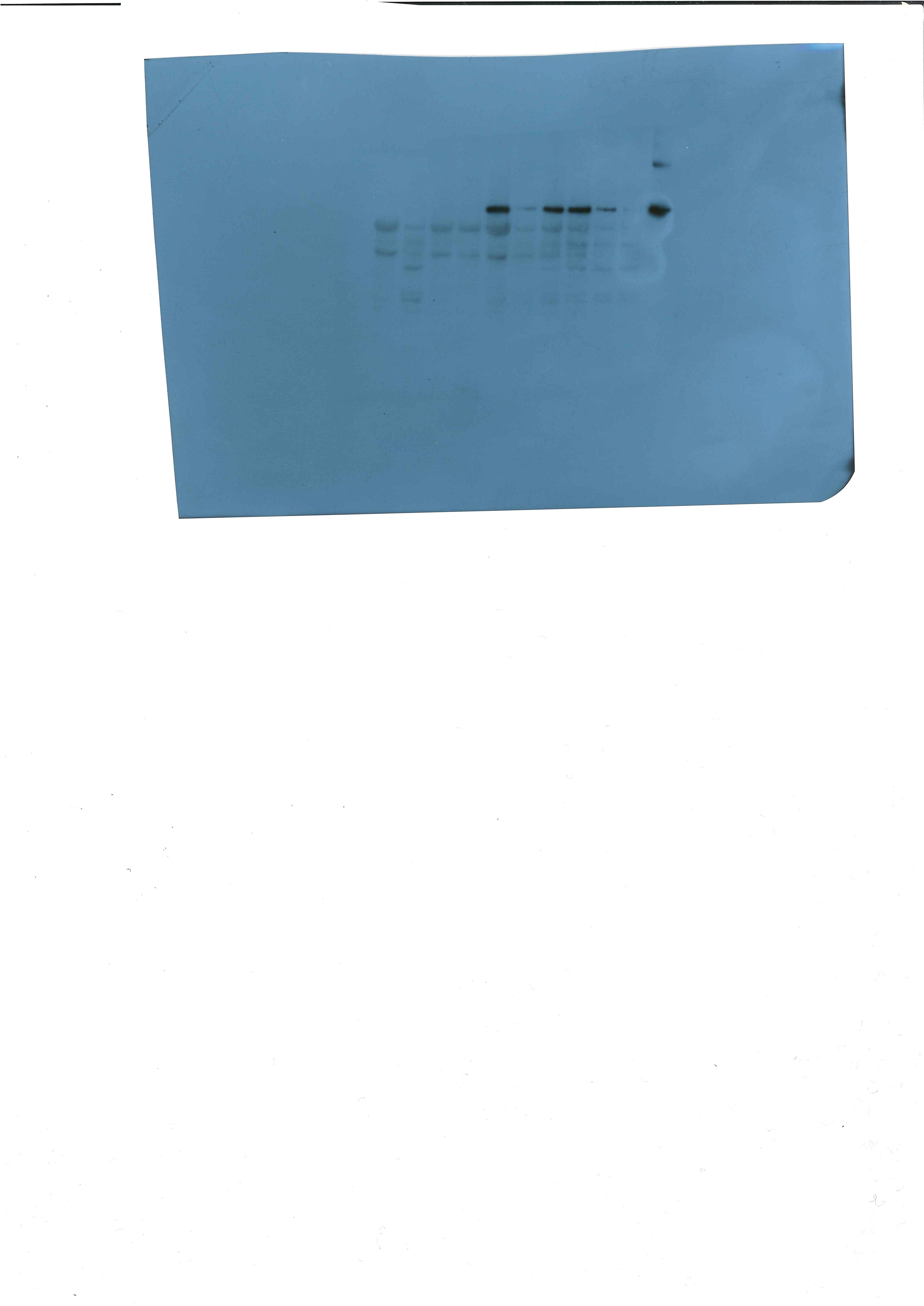

Supplement: Supplemental Information 10 [file peerj-08-8904-s010.zip › Raw data-4 Feb/Fig. 4B. CPS Western Blot.png]

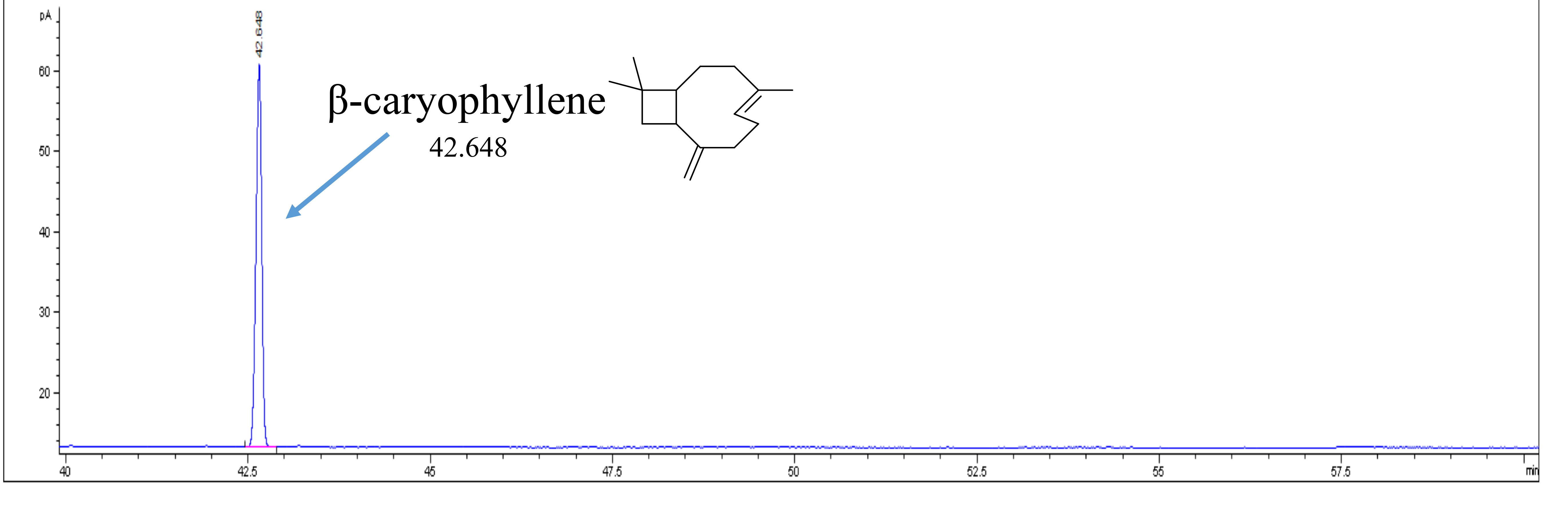

Supplement: Supplemental Information 10 [file peerj-08-8904-s010.zip › Raw data-4 Feb/Fig. 5D Beta-CP.png]

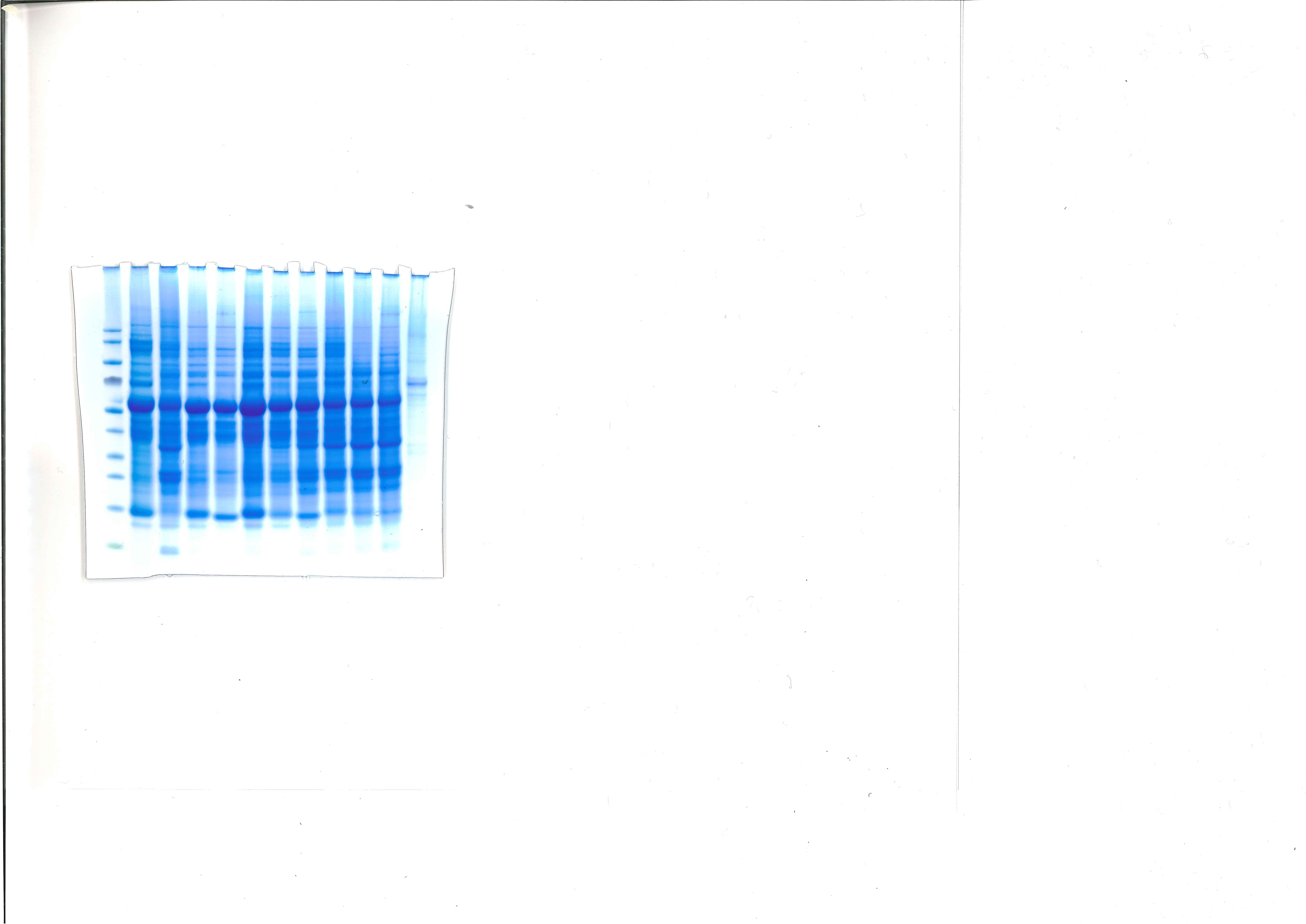

Supplement: Supplemental Information 10 [file peerj-08-8904-s010.zip › Raw data-4 Feb/Fig. 4A.CPS SDS-PAGE.png]

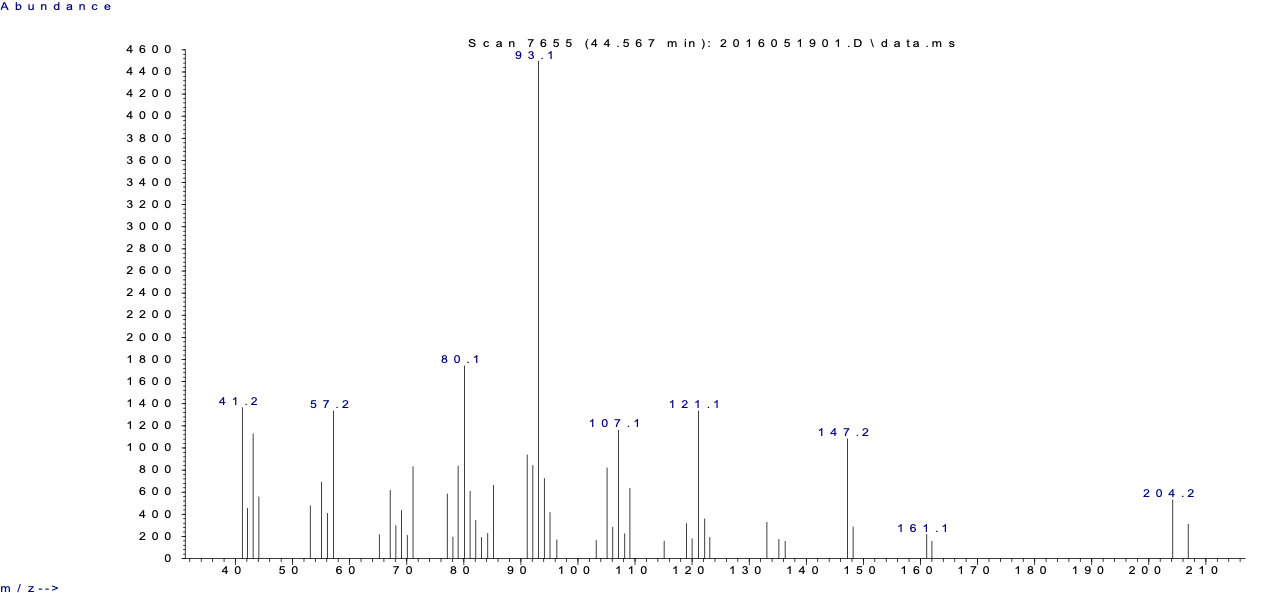

Supplement: Supplemental Information 10 [file peerj-08-8904-s010.zip › Raw data-4 Feb/Fig. 5F. Alpha-humulene.png]

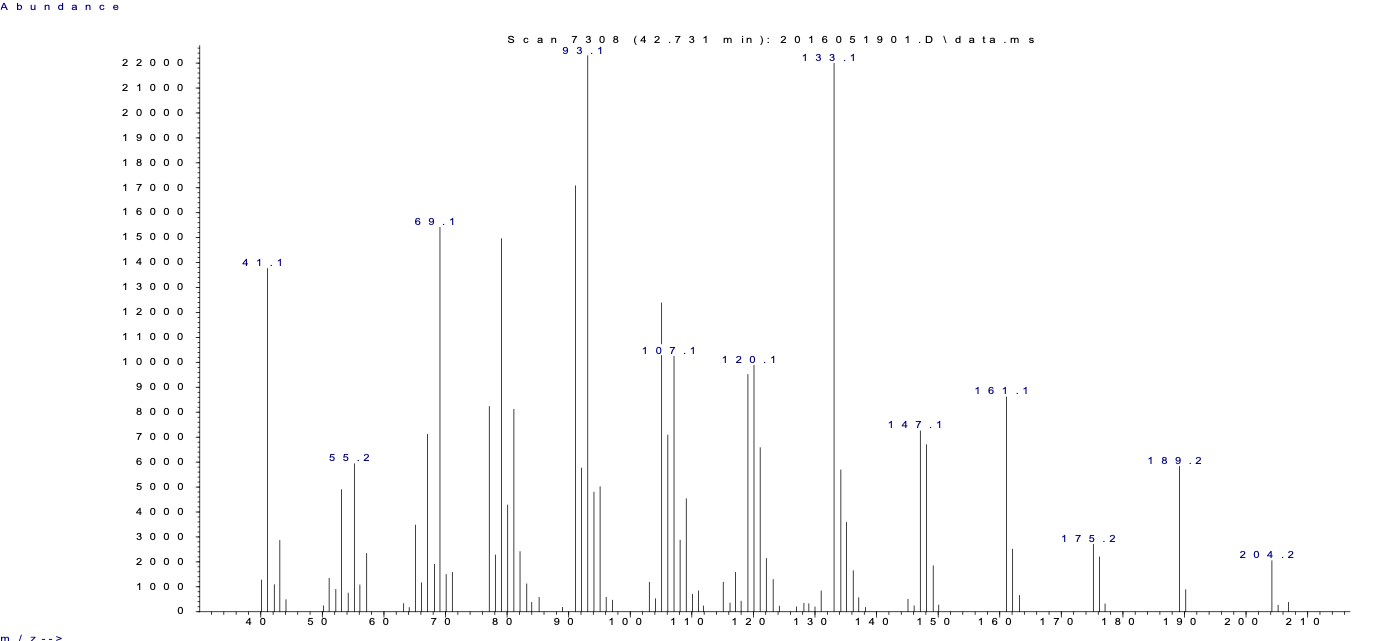

Supplement: Supplemental Information 10 [file peerj-08-8904-s010.zip › Raw data-4 Feb/Fig. 5E. Beta-Caryophyllene.png]

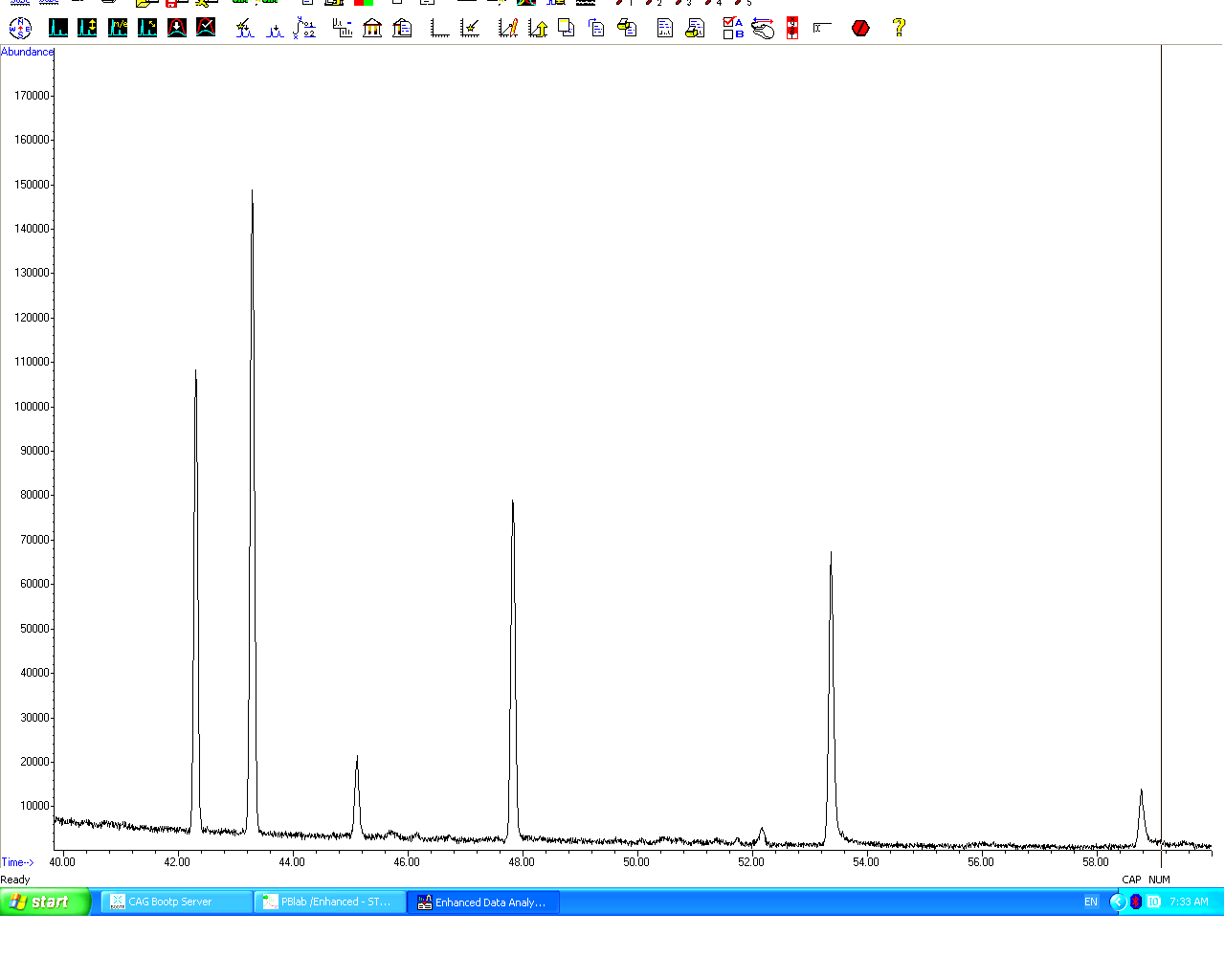

Supplement: Supplemental Information 10 [file peerj-08-8904-s010.zip › Raw data-4 Feb/Fig. 5D. CPS-CHRO.png]

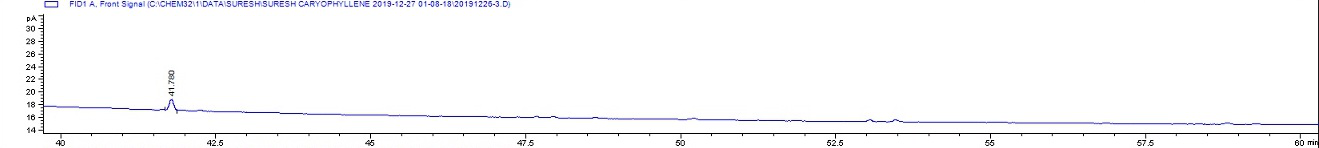

Supplement: Supplemental Information 10 [file peerj-08-8904-s010.zip › Raw data-4 Feb/Fig. 5C. Puri.EV.png]

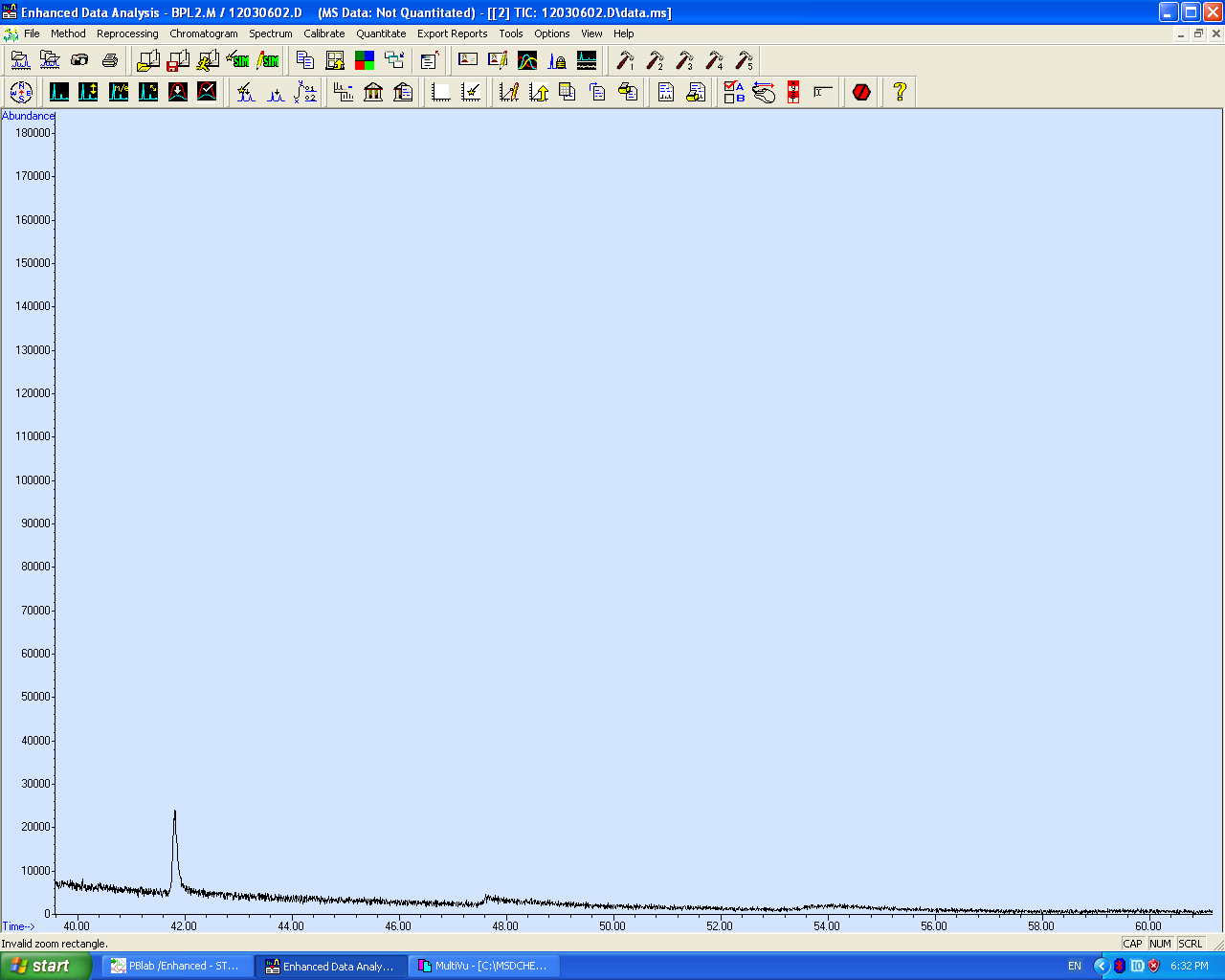

Supplement: Supplemental Information 10 [file peerj-08-8904-s010.zip › Raw data-4 Feb/Fig. 5B Control-NB.png]

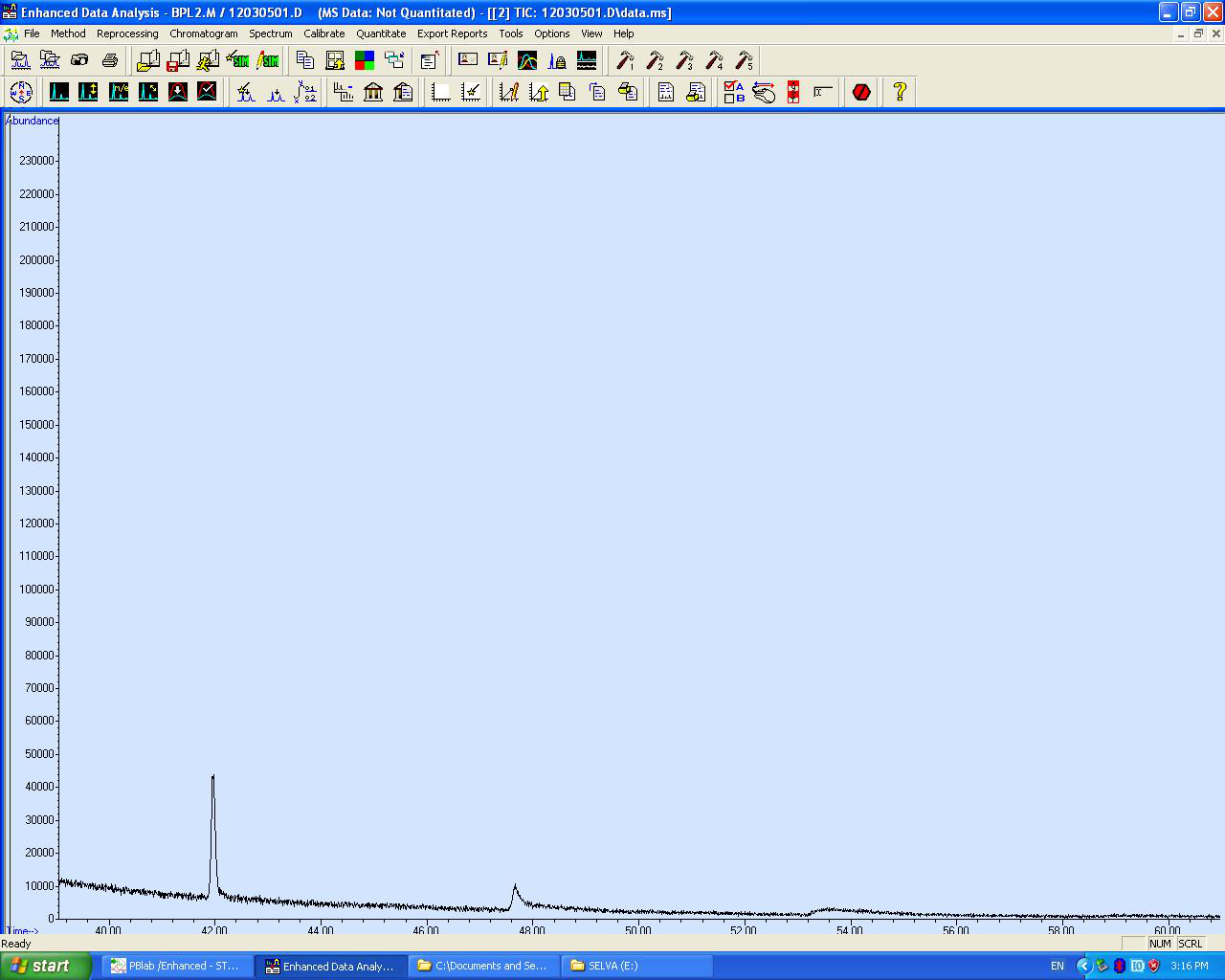

Supplement: Supplemental Information 10 [file peerj-08-8904-s010.zip › Raw data-4 Feb/Fig. 5A Control-EV.png]
