# Supplementary material for: Transient expression and purification of β-caryophyllene synthase in Nicotiana benthamiana to produce β-caryophyllene in vitro
Source: PeerJ. 2020 Apr 28;8:e8904. doi: 10.7717/peerj.8904 (PMC7194099; doi:10.7717/peerj.8904)
Supplement: Supplemental Information 10 [file peerj-08-8904-s010.zip › Raw data-4 Feb/Fig. 3. Relative Expression Results.pdf]

Relative Expression Results

Parameter	Value
Iterations	2000

Gene	Type	Reaction Efficiency	Expression	Std. Error	95% C.I.	P(H1)	Result
EF1	REF	0.9092	1.000				
3 DPI	TRG	0.8857	14.662	12.640 - 16.681	12.149 - 18.880	0.021	UP
6 DPI	TRG	0.8857	159.228	121.899 - 192.428	110.751 - 230.103	0.009	UP
9 DPI	TRG	0.8857	361.668	310.087 - 422.183	292.303 - 462.650	0.021	UP
12 DPI	TRG	0.8857	182.687	151.853 - 206.033	149.204 - 251.332	0.021	UP
15 DPI	TRG	0.8857	1.000	0.840 - 1.199	0.772 - 1.296	0.778	
Actin	REF	0.8438	1.000				

Interpretation

3 DPI is UP-regulated in sample group (in comparison to control group) by a mean factor of 14.662 (S.E. range is 12.640 - 16.681).

3 DPI sample group is different to control group. P(H1)=0.021

6 DPI is UP-regulated in sample group (in comparison to control group) by a mean factor of 159.228 (S.E. range is 121.899 - 192.428).

6 DPI sample group is different to control group. P(H1)=0.009

9 DPI is UP-regulated in sample group (in comparison to control group) by a mean factor of 361.668 (S.E. range is 310.087 - 422.183).

9 DPI sample group is different to control group. P(H1)=0.021

12 DPI is UP-regulated in sample group (in comparison to control group) by a mean factor of 182.687 (S.E. range is 151.853 - 206.033).

12 DPI sample group is different to control group. P(H1)=0.021

15 DPI sample group is not different to control group. P(H1)=0.778

Non-Normalised Results

Gene	Type	Reaction Efficiency	Expression	Std. Error	95% C.I.	P(H1)	Result
EF1	REF	0.9092	1.000	0.941 - 1.063	0.900 - 1.112	0.776	
3 DPI	TRG	0.8857	14.662	12.640 - 18.599	11.465 - 19.342	0.000	UP
6 DPI	TRG	0.8857	159.228	124.687 - 192.428	112.487 - 240.657	0.000	UP
9 DPI	TRG	0.8857	361.668	303.325 - 436.155	291.391 - 483.325	0.000	UP
12 DPI	TRG	0.8857	182.687	140.913 - 218.628	134.067 - 282.948	0.000	UP
15 DPI	TRG	0.8857	1.000	0.791 - 1.271	0.690 - 1.452	0.590	
Actin	REF	0.8438	1.000	0.885 - 1.134	0.832 - 1.202	0.590	
